# Synthesis of (−)−deoxypodophyllotoxin and (−)−epipodophyllotoxin via a multi-enzyme cascade in *E. coli*

**DOI:** 10.1186/s12934-021-01673-5

**Published:** 2021-09-20

**Authors:** Davide Decembrino, Alessandra Raffaele, Ronja Knöfel, Marco Girhard, Vlada B. Urlacher

**Affiliations:** Institute of Biochemistry, Heinrich-Heine University Düsseldorf, Universitätsstraße 1, 40225 Düsseldorf, Germany

**Keywords:** *E. coli*, Podophyllotoxin, Deoxypodophyllotoxin, Epipodophyllotoxin, P450, Plant biosynthetic pathway, Multi-enzyme cascade, *Sinopodophyllum hexandrum*

## Abstract

**Background:**

The aryltetralin lignan (−)−podophyllotoxin is a potent antiviral and anti-neoplastic compound that is mainly found in *Podophyllum* plant species. Over the years, the commercial demand for this compound rose notably because of the high clinical importance of its semi-synthetic chemotherapeutic derivatives etoposide and teniposide. To satisfy this demand, (−)−podophyllotoxin is conventionally isolated from the roots and rhizomes of *Sinopodophyllum hexandrum*, which can only grow in few regions and is now endangered by overexploitation and environmental damage. For these reasons, targeting the biosynthesis of (−)−podophyllotoxin precursors or analogues is fundamental for the development of novel, more sustainable supply routes.

**Results:**

We recently established a four-step multi-enzyme cascade to convert (+)−pinoresinol into (−)−matairesinol in *E. coli*. Herein, a five-step multi-enzyme biotransformation of (−)−matairesinol to (−)−deoxypodophyllotoxin was proven effective with 98 % yield at a concentration of 78 mg/L. Furthermore, the extension of this cascade to a sixth step leading to (−)−epipodophyllotoxin was evaluated. To this end, seven enzymes were combined in the reconstituted pathway involving *inter alia* three plant cytochrome P450 monooxygenases, with two of them being functionally expressed in *E. coli* for the first time.

**Conclusions:**

Both, (−)−deoxypodophyllotoxin and (−)−epipodophyllotoxin, are direct precursors to etoposide and teniposide. Thus, the reconstitution of biosynthetic reactions of *Sinopodophyllum hexandrum* as an effective multi-enzyme cascade in *E. coli* represents a solid step forward towards a more sustainable production of these essential pharmaceuticals.

**Supplementary Information:**

The online version contains supplementary material available at 10.1186/s12934-021-01673-5.

## Background

Herbs and herbal-derived products have a long history as essential components of traditional medical treatments all over the world. Since people health awareness and life expectancy is increasing globally, the demand for effective medicines is also increasing. Nowadays, modern drug development rediscovered plants as a rich source of potent bioactive compounds [1]. In this regard, lignans represent a group of peculiar secondary metabolites with multiple biological activities. In particular, lignans have been described as antioxidant, anti-inflammatory, and powerful cytotoxic compounds, as well as agents preventing the development of breast and prostate cancers or cardiovascular diseases [2, 3]. The aryltetralin lignan (−)−podophyllotoxin from *Podophyllum* plant species has gained much attention due to its potent anti-neoplastic and antiviral properties. There are evidences for its medicinal use through the application of *Podophyllum* species starting back in time to 2,500 BC [4]. (−)−Podophyllotoxin is known for blocking tubulin polymerization and it is currently used as antiviral topical agent. Semisynthetic (−)−podophyllotoxin derivatives like teniposide and etoposide with higher solubility, activity, and lower cytotoxicity are used as chemotherapeutics for the treatment of different cancers [5], with the latter being introduced to the list of essential medicines by the World Health Organization [6, 7].

Conventional isolation of (−)−podophyllotoxin from natural sources like *Podophyllum* and *Sinopodophyllum* plants and other related *Berberidaceace* species [8, 9] has become environmentally unbearable due to unregulated plant uprooting within the few regions where these plants can be cultivated [10]. To overcome these limitations, various organometallic chemical catalysts have been successfully combined with single or multiple enzymatic steps, although such approaches often involve the usage of expensive/toxic reagents and harsh reaction conditions [11, 12]. In addition, because of increasing environmental awareness, researchers are keen on putting aside such cumbersome - though elegant - chemo-synthetic approaches and disclosing the potential of enzymes as biocatalysts. Either alone or combined in multi-enzyme cascades, *in vitro* or *in vivo*, biocatalysts generally allow to achieve reactions under mild, biologically compatible reaction conditions, with minor waste production and lower reagents toxicity in comparison to chemical catalysts [13–15].

Recent advances in metabolic engineering and synthetic biology boosted the production of plant secondary metabolites in recombinant microorganisms, in which partial or entire plant pathways were reconstituted [16–18]. Over the years, the lignan biosynthetic pathways have been explored in plants, and the enzymes involved in the biotransformation of the phenylpropanoid (*E*)-coniferyl alcohol to the intermediate compound (−)−matairesinol **1** have been disclosed [19–21]. Yet, only very recently light was shed on the genome of *Sinopodophyllum hexandrum*, which allowed the elucidation of the subsequent steps in the biosynthetic route to (−)−deoxypodophyllotoxin [22, 23]. In a very recent study, Schultz et al. reported a mg-scale production of (−)−deoxypodophyllotoxin **6**  via an engineered biosynthetic pathway in tobacco leaves [24]. Expression of 16 genes encoding both an engineered coniferyl alcohol pathway and the main (−)−deoxypodophyllotoxin pathway from mayapple resulted in the accumulation of up to 4.3 mg/g dry plant weight (−)−deoxypodophyllotoxin **6** (isolated yield of 0.71 mg/g dry plant weight) [24].

**Fig. 1:**
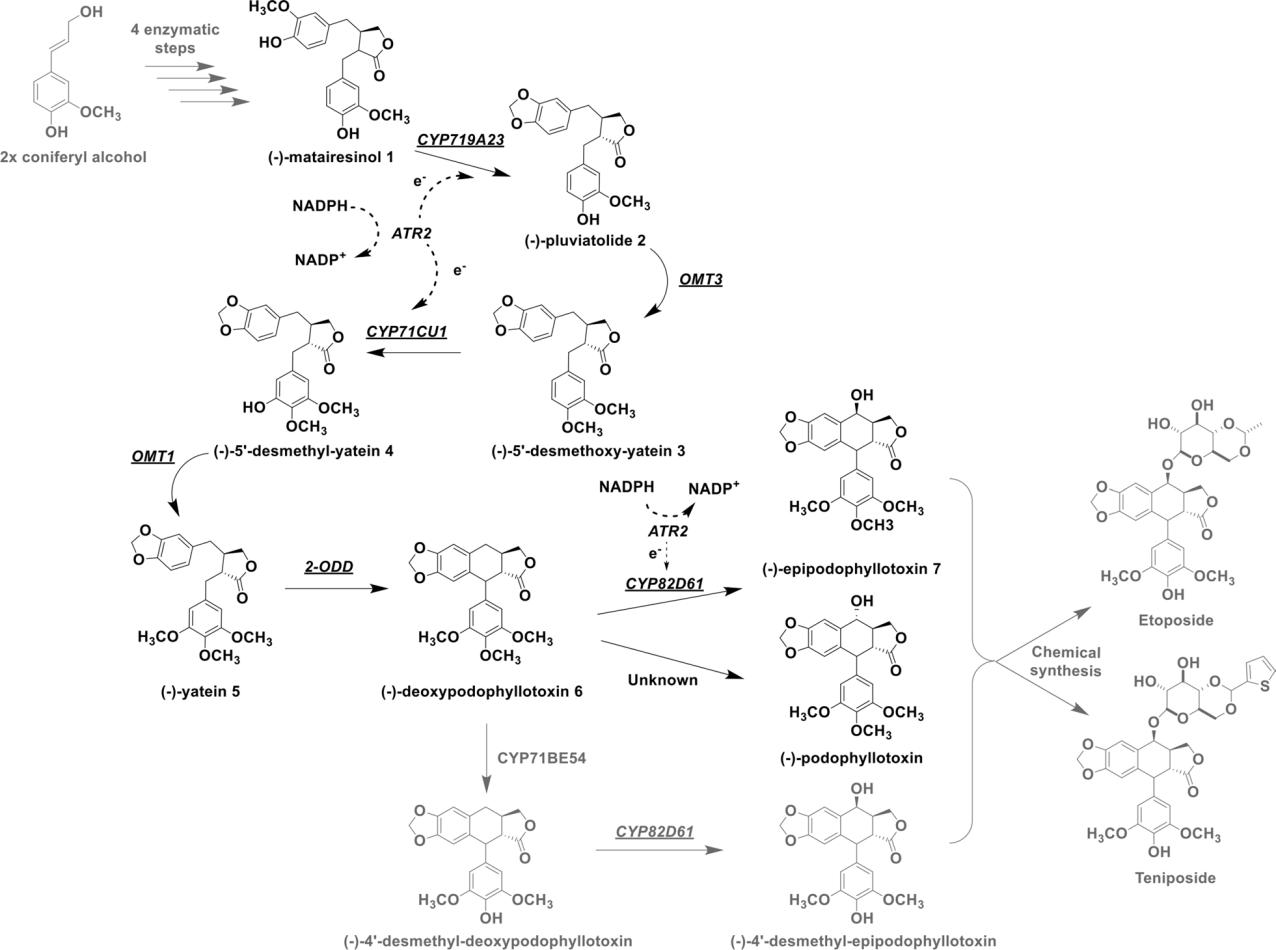
Schematic view for the biosynthesis of (−)−podophyllotoxin and some semi-synthetic derivatives thereof. The six enzymes from *S. hexandrum* that were assembled in this study are underlined. Individual compounds produced in the herein described multi-enzyme cascade are highlighted by a **1** to **7** numbering. Compounds **2**, **3**, **4**, and **7** are not commercially available; (−)−pluviatolide **2** was isolated and identified in our previous work [25]

Against this background, the aim of this study was to reconstitute a part of the *S. hexandrum* pathway in *E. coli* for the first time to allow the biosynthesis of (−)−deoxypodophyllotoxin **6** and (−)−epipodophyllotoxin **7** from the precursor (−)−matairesinol **1** (Fig. 1). In two previous studies we have reported on efficient multi-enzyme cascades in *E. coli* starting from coniferyl alcohol or (+)−pinoresinol to yield (−)−matairesinol **1** [25, 26]. In the subsequent steps of the *S. hexandrum* metabolic pathway, (−)−matairesinol **1** is first converted to (−)−pluviatolide **2** by the cytochrome P450 monooxygenase (P450 or CYP) CYP719A23 [23]. The following methylation of (−)−pluviatolide **2** is catalysed by the (−)−pluviatolide-O-methyltransferase (OMT3) yielding (−)−5’-desmethoxy-yatein **3**, which is in turn hydroxylated to (−)−5’-desmethyl-yatein **4** catalysed by CYP71CU1. Afterwards, a second methylation step is executed by the 5’-desmethyl-yatein O-methyltransferase (OMT1) to produce (−)−yatein **5**. Finally, 2-oxoglutarate/Fe(II)-dependent dioxygenase (2-ODD) catalyses the oxidation of (−)−yatein **5** to (−)−deoxypodophyllotoxin **6**; during this reaction a C-C bond is formed leading to ring closure, which is characteristic for the aryltetralin scaffold of the (−)−podophyllotoxin-like lignans [27]. In this study, we combined these enzymes in a modular manner which allowed to achieve the efficient 5-step conversion of (−)−matairesinol **1** to (−)−deoxypodophyllotoxin **6** with 98 % yield. Whereas the heterologous expression and the reconstitution of CYP719A23 activity in *E. coli* has been described in our previous report, CYP71CU1 has been expressed in *E. coli* for the first time.

To date, the final stereo- and regioselective hydroxylation of (−)−deoxypodophyllotoxin **6** to (−)−podophyllotoxin remains elusive, since the physiological enzyme of *S. hexandrum* catalysing this reaction has yet to be identified [28]. However, another P450 from *S. hexandrum*, namely CYP82D61, has been associated to the synthesis of the epimer (−)−epipodophyllotoxin **7** [23], and this P450 was herein expressed in active state in *E. coli* for the first time as well. Finally, a multi-enzyme cascade for the biosynthesis of (−)−epipodophyllotoxin **7** from (−)−matairesinol **1** was designed by assembling the six enzymes from *S. hexandrum* (involving all three P450s) and the cytochrome P450 reductase (CPR) ATR2 from *Arabidopsis thaliana* in a modular manner exploiting two *E. coli* whole-cell catalysts in one reaction pot.

## Results and discussion

### **Screening of*E. coli*strains to express OMT3, OMT1, CYP71CU1 and 2-ODD**

Following the genome mining efforts which led to their identification [23] the heterologous expression in *E. coli* of the individual genes of the methyltransferases OMT3, OMT1 and the dioxygenase 2-ODD have been previously reported in literature [23, 27]. Differently, the expression of CYP71CU1 was attempted in *Saccharomyces cerevisiae* only [23]. Within this work, we first selected an *E. coli* strain most appropriate for the co-expression of all enzymes to assemble a functional multi-enzyme cascade. To achieve this, we compared the individual expression levels of the *omt3*, *omt1*, and *2-odd* genes in the *E. coli* strains BL21 (DE3), C41 (DE3) and C43 (DE3). In summary, the production of OMT3 (41 kDa), OMT1 (38 kDa) and 2-ODD (35 kDa) using their native gene sequences (see Additional file 1) was successful in all strains as judged by SDS-PAGE (Additional file 1: Figures S1, S2 and S3).

The heterologous expression of CYP71CU1 was endeavoured as well. The physiological bond of the N-terminal moiety to the endoplasmic reticulum membrane, which is a typical feature of eukaryotic P450s, is often a drawback to achieve functional expression in prokaryotic hosts because of the lack of organelles and well-developed inner membranes [29]. Nevertheless, after the expression of the complete *E. coli* codon optimized sequence, named *cyp71cu1oc_wt*, the SDS-PAGE revealed a band in the range of 56 kDa in comparison to the respective negative control (Additional file 1: Figure S4). *E. coli* C41 (DE3) appeared as the most suitable strain for CYP71CU1 expression. For a more detailed investigation, CO-difference spectra were recorded to estimate the amount of soluble P450. After expression in every strain, a peak at 420 nm was seen along with a peak at 450 nm (Additional file 1: Figure S5). Unambiguously, ~ 25 µg_P450_/g_cww_ and ~ 65 µg_P450_/g_cww_ were calculated for *E. coli* BL21 (DE3) and C43 (DE3), respectively, in comparison to ~ 100 µg_P450_/g_cww_ for C41 (DE3). Based on these results, *E. coli* C41 (DE3) was chosen as chassis for all following experiments.

### Optimization of CYP71CU1 expression

Since the intrinsic activity of P450s is generally low in comparison to many other enzymes, P450s involved in multi-enzyme cascades have often been reported as a bottleneck [30]. Therefore, improving and optimizing *cyp71cu1* expression and activity represents a crucial step for the development of an efficient multi-enzyme cascade. We optimized several expression parameters: By prolonging the cultivation time from 20 to 48 h an increase of the expression level (160 µg_P450_/g_cww_ vs. 100 µg_P450_/g_cww_) was achieved. In addition, the *E. coli* codon optimized gene sequence was compared to the *cyp71cu1* native sequence. After 48 h expression, 263 µg_P450_/g_cww_ was estimated with the native sequence, which is ~ 1.5-fold higher than the expression of the codon optimized gene sequence (Additional file 1: Figure S6; Table S8).

Further optimization was attempted by manipulating the membrane associated N-terminal region of CYP71CU1, since the partial or total truncation and/or manipulation by changing the sequence of the putative N-terminal membrane-associated region has often been proven successful in raising the expressions levels of plant P450s in *E. coli* [31]. A proline-rich sequence was identified that followed the first 26 amino acids of CYP71CU1; this is a typical feature of microsomal P450s which is proposed to be crucial for the correct protein folding [32]. Variants with deletion of the first 20 amino acids (Δ20) were generated. In addition, a DNA-triplet encoding for the amino acid alanine was introduced as the second codon within the truncated gene sequence, since the translation efficiency was proposed to be enhanced by this strategy [31, 33].

This manipulation remarkably influenced the amount of detectable soluble CYP71CU1; a 3-fold improvement was observed as compared with the expression levels of the non-truncated versions (Additional file 1: Table S8). Specifically, 813 µg_P450_/g_cww_ were achieved using the manipulated native gene (*cyp71cu1Δ20nat*) and 465 µg_P450_/g_cww_ with the manipulated codon optimized sequence (*cyp71cu1Δ20oc*). Finally, the expression temperature was varied between 20 and 30 °C; the optimum for the expression of CYP71CU1 variants was 25 °C. The results are summarized in Additional file 1:  Table S9; Figure S7.

### Initial enzyme activity tests and initial evaluation of enzyme co-expression

**Fig. 2:**
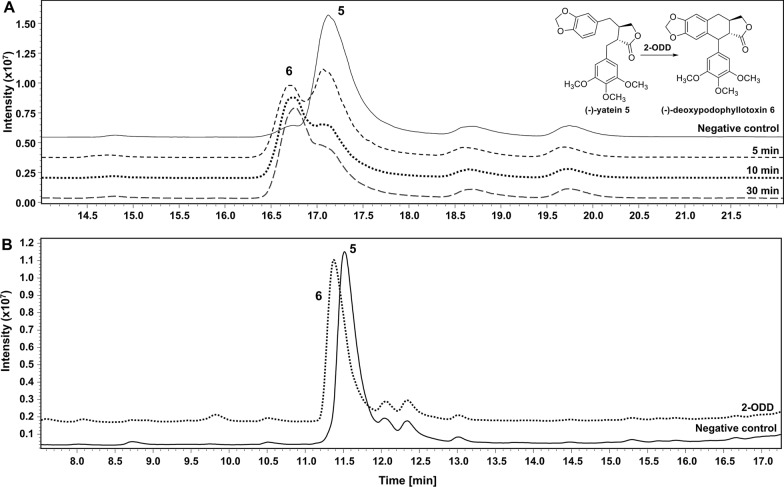
LC/MS analysis to monitor activity of *E. coli* whole cell biocatalysts expressing 2-ODD. **A** (−)−yatein **5** depletion followed over 30 min (analysis method 3). **B** 3 h conversion of (−)−yatein **5** (analysis method 1). Reaction conditions: 25 °C, 1,500 rpm (Eppendorf ThermoMixer C), 2 ml reaction tubes with open lids, 500 µl cell suspension (70 g/L in resuspension buffer), 200 µM (−)−yatein **5**

Since most of the substrates for the individual enzymes are not commercially available, only 2-ODD activity could be tested prior to the multi-enzyme cascade assembly. Due to the high structural similarity of the substrate (−)−yatein **5** and the product (−)−deoxypodophyllotoxin **6**, their behaviour upon chromatographic resolution was very similar resulting in very close retention times (RT) and overlapping peaks (regardless of the separation method used), which made an exact estimation of (−)−yatein **5** conversion by 2-ODD difficult. However, both compounds could unambiguously be distinguished by their characteristic mass fragmentation (m/z) patterns (Additional file 1: Table S6), and by additional spiking of samples with authentic (−)−deoxypodophyllotoxin **6** (Additional file 1: Figure S8A). In order to monitor the reaction progress, (−)−yatein **5** depletion by recombinant *E. coli* cells expressing 2-ODD was followed over time; substrate **5** and product **6** were concomitantly detected as twin-peaks already after 5 min (~ 40 % conversion) and 10 min (~ 65% conversion) (Fig. 2A). Complete conversion of 200 µM (−)−yatein **5** was observed after 3 h (no mass fragments of (−)−yatein **5** were detected at this time point; Fig. 2B).

Next, the activity of OMT3 was evaluated. In our previous work, the OMT3’s substrate (−)−pluviatolide **2** was successfully isolated after production in *E. coli* from (−)−matairesinol **1** by *S. hexandrum* CYP719A23 supported by the cytochrome P450 reductase ATR2 from *Arabidopsis thaliana* [25]. To perform the two-step biotransformation of (−)−matairesinol **1** to (−)−5’-desmethoxy-yatein **3**, CYP719A23 and ATR2 (plasmid pETDuet_atr2_cyp719a∆23oc) were co-expressed with OMT3 (plasmid pCDFDuet_omt3) (Additional file 1: Table S3). Utilizing 200 µM (−)−matairesinol **1**, three major peaks were detected after 30 min. One peak was recognized as (−)−matairesinol **1** (m/z 341 [M + H-H_2_O]^+^, 360 [M + 2 H]^2+^) and one as (−)−pluviatolide **2** (m/z 339 [M + H-H_2_O]^+^, 358 [M + 2 H]^2+^). The predominant fragment ions of the third peak (m/z 372 [M + 2 H]^2+^, 354 [M + 2 H-H_2_O]^2+^) suggested a mass difference (Δm) of + 14 g/mol compared to (−)−pluviatolide **2**, pointing to a methylated product. After 3 h the characteristic m/z pattern of this product distinctly increased accompanied by the full conversion of (−)−pluviatolide **2**, endorsing OMT3 activity (Additional file 1: Figure S9). The observed mass fragments corresponded to those associated to the isolated (−)−5’-desmethoxy-yatein **3** reported elsewhere (Additional file 1: Table S6) [23].

CYP71CU1-mediated hydroxylation of (−)−5’-desmethoxy-yatein **3** produced by OMT3 represents the next reaction step of the cascade. Therefore, we next evaluated the co-expression of CYP71CU1 and OMT3 (plasmid pCDFDuet_omt3_cyp71cu1Δ20nat). Under the established optimized expression conditions for CYP71CU1, when co-expressed with OMT3 in the same *E. coli* cell, CYP71CU1 concentration achieved 240 ± 15 µg_P450_/g_cww_ which is 3-fold lower in comparison to the single expressed CYP71CU1 (Additional file 1: Figure S10A). This is not surprising because in our previous work we obseved a significant reduction of  the P450 concentration when multiple genes were co-expressed from the same expression vector [25].

In this regard it is worth mentioning that P450s require electrons which are ultimately delivered by reduced nicotine amide cofactors NAD(P)H via redox partner proteins [34]. Concerning eukaryotic organisms, the redox equivalents from NAD(P)H are transferred by cytochrome P450 reductases (CPR) [35]. Intuitively, the combination of redox proteins belonging to the same organism represents the method of choice, however, CPRs from *S. hexandrum* are unknown to date. In our previous study, ATR2 was found to support CYP719A23 activity both *in vitro* and *in vivo* [25]. In this respect, in plant natural systems a P450 to CPR ratio of ~ 15:1 has been reported [36, 37]; in other words, the activity of multiple P450s should be sufficiently supported by significantly lower amounts of CPR. Based on these considerations, we assumed that the expression level of ATR2 should be sufficient to sustain the activity of both CYP719A23 and CYP71CU1 under co-expression with OMT3 in one *E. coli* cell (plasmids pETDuet_atr2_cyp719a∆23oc and pCDFDuet_omt3_cyp71cu1Δ20nat).

The inclusion of OMT1 and 2-ODD catalysing the follow-up reaction steps finished the assembly of the desired multi-enzyme cascade. 2-ODD and OMT1 were co-expressed utilizing the plasmid pCOLADuet_2-odd_omt1 (Additional file 1: Table S3; Figure S10B).

In summary, three plasmids were designed in a modular fashion to allow the co-expression of all necessary genes and to target the biosynthesis of (−)−deoxypodophyllotoxin **6** in one *E. coli* cell (Additional file 1: Figure S11).

### **Implementation of the cascade from (−)−matairesinol 1 to (−)−deoxypodophyllotoxin 6 in*E. coli***

#### One-cell approach

**Fig. 3:**
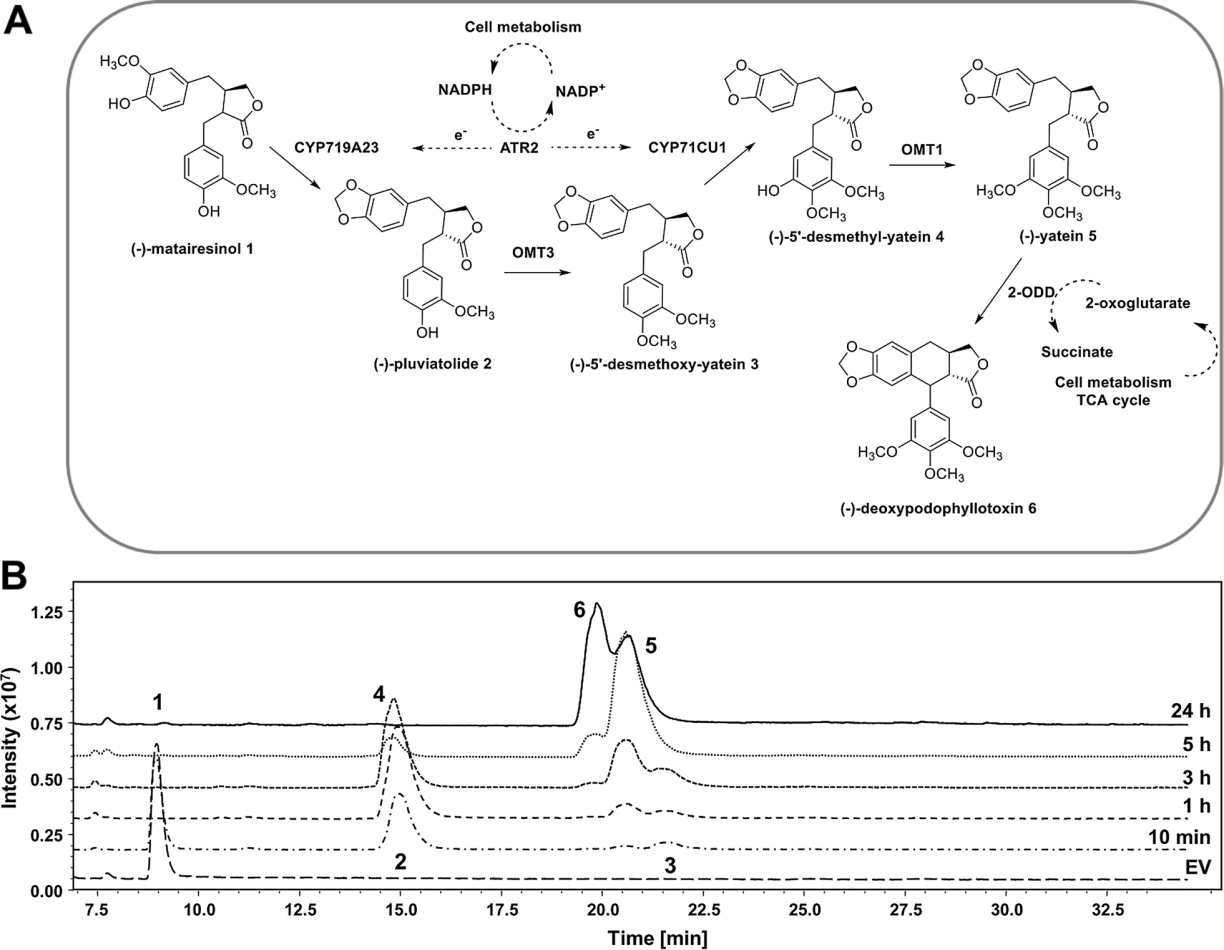
** A** Schematic picture of the one-cell biotransformation of (−)−matairesinol **1** to (−)−deoxypodophyllotoxin **6**. **B** LC/MS analysis (method 4) at various time points of the reaction. Reaction conditions: 25 °C, 250 rpm (Orbital shaker), baffled Erlenmeyer flasks, 10 mL cell suspension, 200 µM (−)−matairesinol **1**

In the first approach to achieve the 5-step biotransformation of (−)−matairesinol **1** to (−)−deoxypodophyllotoxin **6**, all necessary pathway enzymes were co-expressed in a single cell harbouring three plasmids (Fig. 3A). This one-cell approach was conducted in flasks with 10 mL cell suspension and 200 µM (−)−matairesinol **1**. Following the reaction over time, substrate depletion, intermediate formation and consumption, as well as the final product (−)−deoxypodophyllotoxin **6** were monitored. In the absence of commercially available authentic references, the identification of the intermediates (−)−5’-desmethoxy-yatein **3** and (−)−5’-desmethyl-yatein **4** was possible relying on the work of Lau and Sattely (Additional file 1: Table S6) [23].

The activity of CYP719A23 in combination with ATR2 was proven by ~ 50 % consumption of (−)−matairesinol **1** (m/z 341 [M + H-H_2_O]^+^, 359 [M + H]^+^) within the first 10 min, and formation of the intermediate (−)−pluviatolide **2** (m/z 339 [M + H-H_2_O]^+^, 358 [M + H]^+^), which accounted for 86 % of all detected intermediates at that time point. Complete (−)−matairesinol **1** depletion was observed after 1 h; at that time point the presence of the intermediates (−)−5’-desmethoxy-yatein **3** (~ 7 %, m/z 353 [M + H-H_2_O]^+^, 371 [M + H]^+^) and (−)−yatein **5** (~ 13 %, m/z 383 [M + H-H_2_O]^+^, m/z 401 [M + H]^+^) was observed, confirming the activities of OMT3 and OMT1, respectively. The activity of CYP71CU1 can also be concluded, given that this enzyme performes the hydroxylation step between the steps catalysed by OMT3 and OMT1. After 3 h, (−)−5’-desmethyl-yatein **4** formed in the CYP71CU1-catalysed reaction could be better evaluated. The structural similarity of (−)−5’-desmethyl-yatein **4** to (−)−pluviatolide **2** generates overlapping peaks, however distinctive fragmentation patterns allowed to discern the compounds qualitatively. MS signals of (−)−pluviatolide **2** (m/z 339 [M + H-H_2_O]^+^ and 358 [M + H]^+^) were poorly detectable, while the intensity of (−)−5’-desmethyl-yatein **4** fragments (m/z 369 [M + H-H_2_O]^+^ and m/z 387 [M + H]^+^) fragments increased. Notwithstanding the difficult quantification, this finding demonstrates that CYP71CU1 is catalytically active in *E. coli* and it also implies that ATR2 is performing as adequate electron shuttle to both P450s, CYP719A23 and CYP71CU1, within this reaction setup. After 5 h, (−)−5’-desmethyl-yatein **4** (~ 7 %) was detected together with ~ 84 % of the intermediate (−)−yatein **5** (m/z 383 [M + H-H_2_O]^+^, m/z 401 [M + H]^+^). At the same time point, (−)−deoxypodophyllotoxin **6** (m/z 399 [M + H]^+^, 421 [M + Na]^+^) accounts for ~ 10 %. After prolongation of the reaction to 24 h, (−)−deoxypodophyllotoxin **6** and (−)−yatein **5** were detected in a ~ 60:40 ratio, which revealed the 2-ODD-catalysed reaction as an apparent bottleneck (Fig. 3B). This outcome was somewhat unexpected since, as described in the previous chapter, single expressed 2-ODD was able to completely convert 200 µM (−)−yatein **5** within 3 h.

The increased metabolic burden determined by the co-expression of multiple enzymes from three plasmids within a single cell may represent a straightforward explanation for the observed results. The presence of several redox enzymes in the multi-enzyme cascade is also likely to generate competition for cofactors and co-substrates within the cell [38]. Although in all cascade reactions glucose (500 mM) was present in the resuspension buffer as energy and carbon source to support *E. coli* metabolism for TCA cycle and pentose phosphate pathway, its provision likely needs optimization to ensure a good balance between the host primary metabolic machinery and the heterologous one.

Another factor limiting the performance of 2-ODD activity might be availability of the co-substrate 2-oxoglutarate that is required within the TCA cycle [39]. In order to prove this hypothesis, the experiment was repeated with 2-oxoglutarate added in the culture medium, either upon addition of (−)−matairesinol **1** or 4 h thereafter. Indeed, the addition of 2-oxoglutarate lead to a slight improvement of the (−)−deoxypodophyllotoxin **6** to (−)−yatein **5** ratio, but complete conversion of (−)−yatein **5** was never achieved **(**Additional file 1: Figure S8B).

#### Two-cell approach

**Fig. 4:**
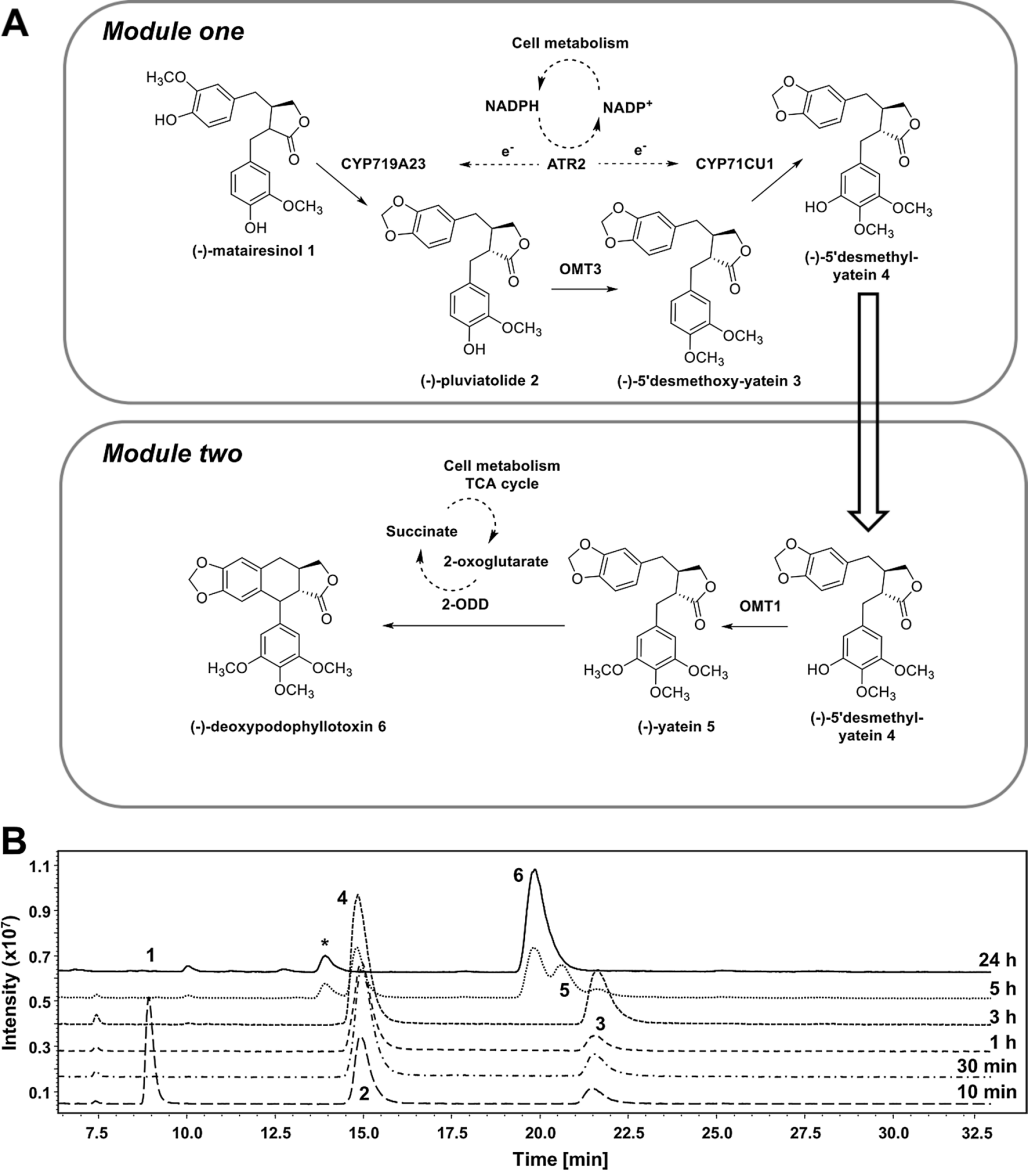
** A** Schematic picture of the sequential two-cell biotransformation of (−)−matairesinol **1** to (−)−deoxypodophyllotoxin **6**. After 3 h conversion by module one, module two was added to the reaction. **B** LC/MS analysis (method 4) at various time points of the reaction. Reaction conditions: 25 °C, 250 rpm (Orbital shaker), baffled Erlenmeyer flasks, 2 × 10 mL cell suspension, 200 µM (−)−matairesinol **1**

It is known for some time that distributing the work of complex biological systems across multiple cells or cell types can improve both efficiency and viability, largely through reducing the metabolic burden on individual cell types (recently reviewed by Grandel et al.) [40]. Following this strategy, the cascade was divided between two *E. coli* cells, which from now on will be referred to as modules. Module one contained CYP719A23 and ATR2 together with OMT3 and CYP71CU1 covering (−)−matairesinol **1** biotransformation to (−)−5’-desmethyl-yatein **4**, while module two harboured OMT1 and 2-ODD to finalize (−)−deoxypodophyllotoxin **6** synthesis (Fig. 4A). The two modules were combined in a sequential manner: 3 h after conversion of 200 µM (−)−matairesinol **1** by module one, module two was added. ~ 60 % (−)−matairesinol **1** depletion was observed within 10 min, and complete (−)−matairesinol **1** conversion after 1 h; (−)−pluviatolide **2** (85 %) and (−)−5’-desmethoxy-yatein **3** (15 %) were the only detectable compounds at this time point. Prior to the addition of module two (after 3 h) no mass fragments related to (−)−pluviatolide **2** were observed; instead, m/z 369 [M + H-H_2_O]^+^ and m/z 387 [M + H]^+^ were recorded for the peak at RT = 14.9 min, identifying (−)−5’-desmethyl-yatein **4** (the final product of module one) as the most prominent product.

Already 2 h after module two was added (corresponding to 5 h total reaction time), the two-cell approach seemed more efficient than the one-cell approach. At this time point, (−)−deoxypodophyllotoxin **6** and (−)−yatein **5** corresponded to ~ 40 % and ~ 23 % of all detected compounds, respectively, and after 24 h (−)−yatein **5** was completely converted to (−)−deoxypodophyllotoxin **6** (Fig. 4B). Obviously, 2-ODD activity was substantially enhanced in the sequential two-cell approach.

It should be noted that a distinct though minor side product peak was detected after the addition of module two at RT = 13.5 min (marked with an asterisk within Fig. 4B). The mass fragments (m/z 385 [M + H]^+^ and 407 [M + Na]^+^) suggest that aryltetralin ring closure may occur before the 2-ODD catalysed oxidation. As (−)−5’-desmethyl-yatein **4** molecular weight is 386 g/mol, the observed difference in mass-to-charge ratio is coherent to a compound with a predicted molecular weight of 384 g/mol, which corresponds to (−)−5’-desmethyl-deoxypodophyllotoxin (Additional file 1: Figure S12).

Application of the two-cell approach was remarkably effective: 196 µM of (−)−deoxypodophyllotoxin **6** (corresponding to 78 mg/L) were quantified as a result of this 5-step enzymatic reaction cascade, corresponding to 98 % theoretical yield unambiguously demonstrating the advantage of this setup in comparison to the one-cell approach.

### Evaluation of (−)−deoxypodophyllotoxin 6 hydroxylation by CYP82D61

In a study by Lau and Sattely, the accumulation of (−)−epipodophyllotoxin **7** was described after (−)−deoxypodophyllotoxin **6** transformation catalysed by CYP82D61 from *S. hexandrum.* Within the same work, this compound was isolated and identified after heterologous expression of CYP82D61 in *N. benthamiana* leaves [23]. Therefore, we attempted the expression of CYP82D61 in *E. coli*. Similar to the optimization strategy applied for CYP71CU1, the N-terminal membrane-associated sequence of CYP82D61 was manipulated by generating a truncated variant where the first 23 amino acids were deleted (Δ23) and the second codon was substituted by alanine (see Additional file 1). Using *E. coli* C41(DE3) as host, the expression of the *E. coli* codon optimized gene sequence *cyp82d61oc_wt* and the manipulated gene variant *cyp82d61Δ23oc* was conducted for 48 h at temperatures between 20° to 30 °C. Expression of the *wt* gene was successful only at 30 °C and reached 250 µg_P450_/g_cww_. The N-terminal manipulation was beneficial and improved the expression of CYP82D61 at 30 °C resulting in 1,395 µg_P450_/g_cww_ (compared to 936 µg_P450_/g_cww_ at 25 °C, and no detectable expression at 20 °C.) The results are summarized in Additional file 1: Table S10. Regarding the expression temperature, this outcome was somehow unexpected since multiple physiological features including gene expression have been reported to be enhanced in *S. hexandrum* at temperatures lower than 25 °C [28, 41, 42].

**Fig. 5:**
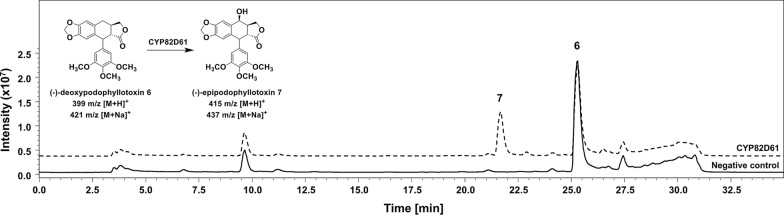
LC/MS analysis (method 2) of CYP82D61 activity for (−)−deoxypodophyllotoxin **6** hydroxylation in comparison to the respective negative control. Reaction conditions: 25 °C, 1,500 rpm (Eppendorf ThermoMixer C), 2 ml reaction tubes with open lids, 500 µL reaction volume

CYP82D61 activity was further evaluated using recombinant *E. coli* cells co-expressing ATR2 (plasmid pETDuet_atr2_cyp82d61Δ23oc; Additional file 1: Table S3). (−)−Deoxypodophyllotoxin **6** was converted to ~ 30 % after 24 h (Fig. 5). The mass fragments of the product (m/z 415 [M + H]^+^ and m/z 437 [M + Na]^+^) match the features of (−)−epipodophyllotoxin **7** described previously (Additional file 1: Table S6) [23].

### Extension of the multi-enzyme cascade toward (−)−epipodophyllotoxin 7

The established two-cell approach allowed a straightforward extension: For the six-step multi-enzyme cascade from (−)−matairesinol **1** to (−)−epipodophyllotoxin **7**, module one harboured CYP719A23, ATR2, OMT3, and CYP71CU1, whereas module two contained OMT1, 2-ODD, CYP82D61, and ATR2 (Additional file 1: Figure S13). Using this reaction setup, (−)−matairesinol **1** was readily consumed by CYP719A23 within the first 10 min, with (−)−pluviatolide **2** accumulating to its maximum concentration after 1 h and being completely depleted after 3 h. Concomitantly, (−)−5’-desmethoxy-yatein **3** was efficiently hydroxylated by CYP71CU1 resulting in (−)−5’-desmethyl-yatein **4** accumulation to ~ 70 % after 3 h. After 5 h total reaction time (2 h after addition of module two), (−)−5’-desmethyl-yatein **4** was depleted by 50 % through OMT1 yielding (−)−yatein **5**, which soared as the major intermediate at this time point (~ 55 %).

**Fig. 6:**
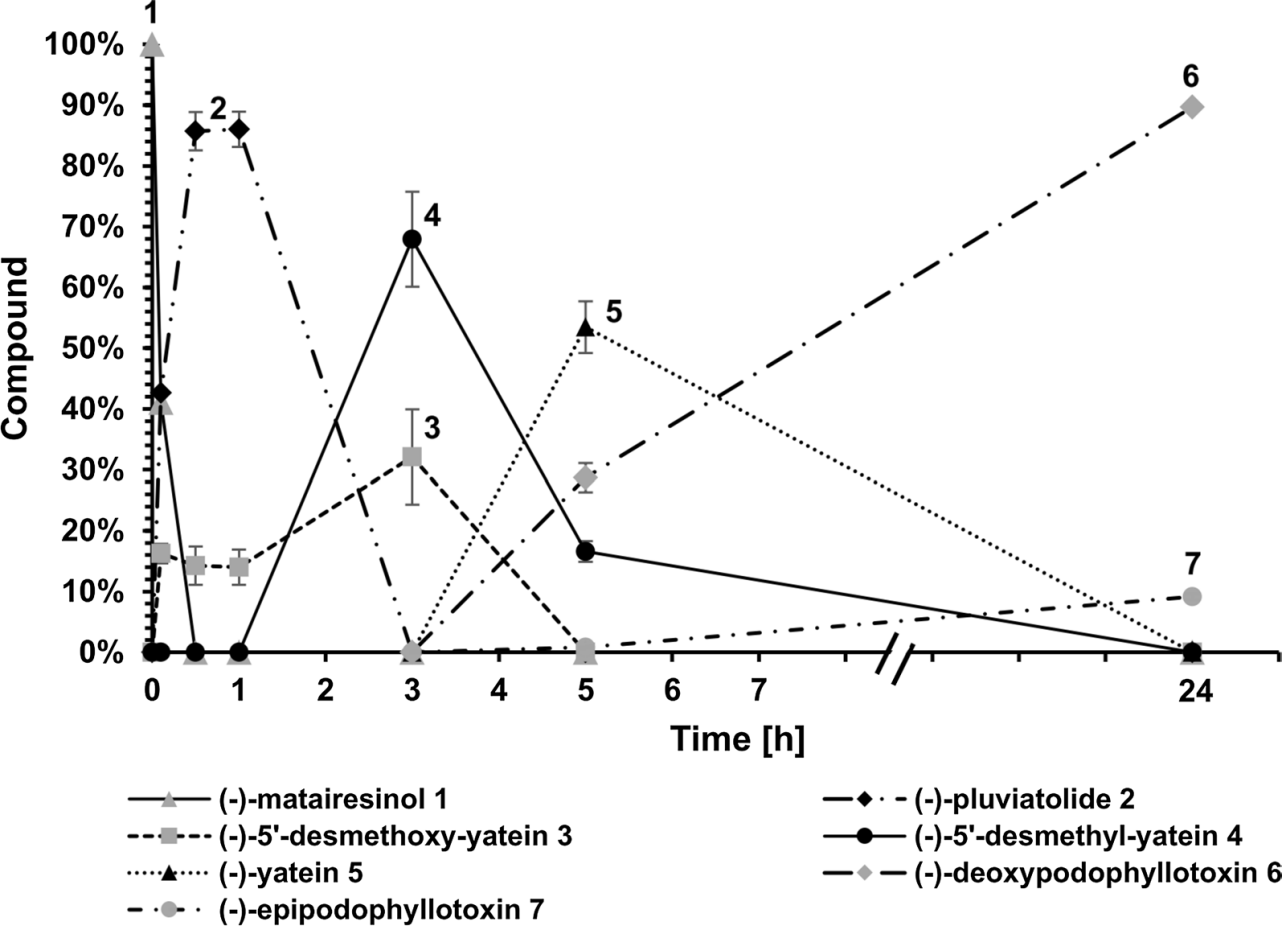
LC/MS analysis (method 4) at various time points of the two-cell biotransformation of (−)−matairesinol **1** to (−)−epipodophyllotoxin **7**. After 3 h conversion by module one, module two was added. Reaction conditions: 25 °C, 250 rpm (Orbital shaker), baffled Erlenmeyer flasks, 2 × 10 mL cell suspension, 200 µM (−)−matairesinol **1**

In comparison to the previous five-step cascade, the inclusion of CYP82D61 and ATR2 into module two appeared to slightly influence the efficacy of 2-ODD, since the turnover of (−)−yatein **5** was slowed down. As hypothesised for the one-cell approach, the addition of redox enzymes may lead to resources competition that reduced enzymatic performance. Nevertheless, full depletion of (−)−yatein **5** by 2-ODD was observed after 24 h, and 160 µM (−)−deoxypodophyllotoxin **6** were quantified being the most abundant product (> 85 %). Additionally, (−)−epipodophyllotoxin **7** (~ 10 %, corresponding to 8.3 mg/L) was observed as the product of (−)−deoxypodophyllotoxin **6** hydroxylation catalysed by CYP82D61 (Fig. 6).

## Conclusions

Within this study, seven plant enzymes were successfully combined to produce the direct precursor of (−)−podophyllotoxin, (−)−deoxypodophyllotoxin **6**, as well as (−)−epipodophyllotoxin **7** in *E. coli*. The described results demonstrate the advantages of a sequential two-cell approach. The hypothesised metabolic burden resulting from the combination of multiple redox enzymes in one *E. coli* cell was relieved in a pragmatic manner, as no cumbersome host manipulation was required. Although P450-mediated reactions are often reported as rate-limiting step in reconstituted biosynthetic pathways, their activity does not represent a bottleneck in the described multi-enzyme cascade.

Due to the modular nature of the developed cascade, it can be extended towards another precursor of etoposide and teniposide by introducing CYP71BE54 from *S. hexandrum* (Fig. 1) [23]. To ensure the utilization of cheaper substrates than (−)−matairesinol **1**, the cascade can also be extended backwards and coupled with, for instance, a four-step cascade from (+)−pinoresinol to (−)−pluviatolide **2** and even further with a two-step conversion of the cheap precursor eugenol to yield pinoresinol; both cascade reactions have already been established in earlier studies by our group [25, 43, 44].

In conclusion, the present study describes the first example of a completely biosynthetic route in *E. coli* to (−)−deoxypodophyllotoxin **6** and (−)−epipodophyllotoxin **7** and represents a solid step forward to the development of a more sustainable production for these direct precursors to etoposide and teniposide.

## Materials and methods

### ***E. coli*strains, enzymes and authentic reference compounds**

*E. coli* DH5ɑ (Clonetech, USA) was used for cloning purposes and *E. coli* BL21 (DE3), C41 (DE3) and C43 (DE3) (Lucigen, USA) served for gene expressions and biotransformations. Endonucleases NcoI, NotI, NdeI and XhoI, Phusion High Fidelity DNA polymerase, thermosensitive alkaline phosphatase (FastAP™), and T4 DNA-ligase were purchased from Thermo Scientific (Germany). T4 polynucleotide kinase and DpnI were purchased from New England Biolabs (USA). Authentic reference compounds used for LC/MS identification and their respective suppliers are listed in Additional file 1: Table S6.

### Genes, plasmids and cloning procedures

Synthetic genes were ordered from BioCat (Germany). (−)−Pluviatolide-O-methyltransferase (GenBank KT390157.1, OMT3), 5’-desmethyl-yatein O-methyltransferase (GenBank KT390155.1, OMT1) and the 2-oxoglutarate/Fe(II)-dependent dioxygenase (deoxypodophyllotoxin synthase, GenBank KT390173.1, 2-ODD) were purchased as native sequences. The gene encoding for CYP71CU1 (GenBank KT390172.1) was ordered as native and *E. coli* codon optimized gene sequences, whereas the gene encoding for CYP82D61 (GenBank KC110995.1) was ordered in *E. coli* codon optimized form only (Additional file 1: Table S1).

With the aim of developing a multi-enzyme cascade, conventional cloning methods were used to insert the sequences of OMT3 and CYP71CU1 in pCDFDuet-1 (Novagen, Merck, Germany) in the multiple cloning sites (MCS) I and II, respectively (Additional file 1: Tables S2, S3; Figures S10, S12). In the same fashion, the sequences of 2-ODD and OMT1 were inserted in MCS I and II of the pCOLADuet-1 vector (Novagen, Merck, Germany), whereas the CYP82D61 sequence was cloned into MCS II of the pETDuet-1 vector (Novagen, Merck, Germany). The sequence coding for the NADPH-cytochrome P450 reductase 2 from *Arabidopsis thaliana* (Protein ID NP_194750.1, ATR2) was manipulated as described elsewhere, [45–47], and cloned in MCS I of the pETDuet-1 vector already harbouring *cyp82d61* (Additional file 1: Tables S2, S3; Figure S12). The genes encoding for CYP719A23 and ATR2 were cloned in the pETDuet-1 vector as described in a previous study by our group (Supplementary Tables S2 and S3; Supplementary Figures S10 and S12) [25]. For the expression of individual enzyme sequences coding for OMT3, CYP71CU1, OMT1 and 2-ODD were cloned in pCDFDuet-1, whereas pETDuet-1 was used for CYP82D61, as summarized in the Additional file 1: Table S3.

The putative membrane associated N-terminal sequences of CYP71CU1 and CYP82D61 were identified using the TMHMM software (version 2.0; http://www.cbs.dtu.dk/services/TMHMM/) in combination with the predictor of signal peptides and membrane protein topology named Spoctopus (https://octopus.cbr.su.se/index.php?about=SPOCTOPUS). Truncated variants of both enzymes were created using the oligonucleotides described in the Additional file 1: Table S4 following the supplier instructions of the Q5 site-directed mutagenesis kit (New England Biolabs, USA).

The correct insertion of genes within the expression vectors was verified via automated Sanger sequencing (Eurofins Genomics GmbH, Germany).

### **Expression of individual genes in*E. coli***, **initial enzyme activity determination, determination of P450 concentration**

The conditions for expression of individual genes in *E. coli* are described in the Additional file 1 (Additional file 1: Methods part).

The activities of individual enzymes for which substrates are commercially available were tested in in 2 mL reaction tubes in a reaction volume of 500 µL composed by the 70 g/L recombinant *E. coli* cell suspension with 200 µM substrate dissolved in DMSO (final DMSO concentration 2 % (v/v)). Reaction tubes were incubated with open lids at 25 °C, 1,500 rpm (ThermoMixer C, Eppendorf, Germany) and samples for LC/MS analysis were taken at selected time points. Since (−)−pluviatolide **2** is not commercially available, OMT3 activity was screened via a two-step enzymatic cascade starting from (−)−matairesinol **1**, with co-expression of ATR2 to sustain the activity of CYP719A23.

For determination of P450 concentration, thawed cell pellets were resuspended in 5 mL 50 mM potassium phosphate buffer (pH 7.5) supplemented with 100 µM phenylmethylsulfonylfluoride (PMSF). Cell disruption was done via sonication (Sonifier 250, Branson, USA), and soluble and insoluble protein fractions were separated by centrifugation (40,000 x g, 25 min, 4 °C). P450 concentrations were calculated by recording CO-difference spectra as described elsewhere with extinction coefficient ε_450nm_ = 91 mM^− 1^ cm^− 1^ [48].

### **Co-expression of genes in*E. coli*and biotransformation conditions**

Co-expression of genes in *E. coli* for use as whole-cell biocatalysts was carried out as follows: Prior to expression of the target genes, biological duplicates of *E. coli* cells transformed with the respective plasmid combinations were precultured in 5 mL Luria-Bertani (LB) medium supplemented with the appropriate antibiotics (streptomycin 50 µg/mL and/or ampicillin 100 µg/mL and/or kanamycin 30 µg/mL) and incubated for 16 h at 37 °C, 180 rpm. 50 mL Terrific broth (TB) media supplemented with the appropriate antibiotics were inoculated with 500 µL from the precultures, and cells were grown at 37 °C, 180 rpm to an OD_600_ of 0.6. The expression of target genes was induced with the addition of 0.5 mM isopropyl β-D-1-thiogalactopyranoside (IPTG), and in cases when P450 was co-expressed, cultures were supplemented with 0.5 mM 5-aminolevulinic acid (5-ALA) and 0.1 mM FeSO_4_. Thereafter, the cultures were incubated at 25 °C, 120 rpm for up to 48 h. This reaction setup presents a compromise of the most suitable expression conditions identified for each individually expressed gene.

After co-expression of the respective genes, the *E. coli* cells were harvested by centrifugation (30 min, 3,220 x g, 4 °C) and frozen at −20 °C. Prior to their use as whole-cell biocatalysts, the cell wet weight (cww) was adjusted to 70 g/L (corresponding to ~ 15 g/L cell dry weight) in resuspension buffer (80 % 50 mM K_2_HPO_4_, 20 % 50 mM KH_2_PO_4_, pH 7.5, 500 mM D-glucose, 0.1 mM IPTG). This freeze-thaw cycle should ease mass transfer across the individual modules’ membranes by increasing cell wall permeability [49].

Biotransformations for synthesis of (−)−deoxypodophyllotoxin **6** starting from (−)−matairesinol **1** in the one-cell approach were carried out in 100 mL Erlenmeyer baffled flasks filled with 10 mL of 70 g/L *E. coli* cell suspension and 200 µM substrate, incubated at 25 °C, 250 rpm in orbital shaker (Multitron, Infors HT, Germany). In a modified setup, 2.5 mM 2-oxoglutarate was added, either upon substrate addition or 4 h thereafter. 500 µL aliquots were taken at selected time points to determine substrate depletion, intermediate formation and consumption, and product concentration over time.

In the two-cell approach, 100 mL Erlenmeyer baffled flasks filled with 10 mL of 70 g/L *E. coli* C41 (DE3) co-expressing CYP719A23, ATR2, OMT3 and CYP71CU1 were employed as module one. After 3 h conversion, 10 mL of 70 g/L *E. coli* C41 (DE3) co-expressing OMT1 and 2-ODD (module two) were added to convert (−)−5’-desmethyl-yatein **4** to (−)−deoxypodophyllotoxin **6**. Later, module two was further enhanced by additional co-expression of CYP82D61 and ATR2 (together with OMT1 and 2-ODD) to extend the reaction towards (−)−epipodophyllotoxin **7**.

### Metabolite analysis

Prior to metabolite extraction 200 µM (+)-sesamin was added as internal standard (IS) to the reaction solution. Metabolites were extracted twice with 1 mL ethyl acetate. The organic phases from the two extractions were combined and evaporated. The residues were resuspended in 50 µl methanol prior to analysis.

The biotransformations of (−)−matairesinol **1** to (−)−deoxypodophyllotoxin **6** or (−)−epipodophyllotoxin **7** - through the intermediates (−)−pluviatolide **2**, (−)−5’-desmethoxy-yatein **3**, (−)−5’-desmethyl-yatein **4** and (−)−yatein **5** - were analysed via liquid chromatography coupled with mass spectrometry (LC/MS) on an LCMS-2020 system (Shimadzu, Japan) equipped with a Chromolith® Performance RP-18e column (100 × 4.6 mm, Merck, Germany). The column temperature was kept at 30 °C and 1 µL sample was injected. Separation was carried out with a mobile phase gradient constituted of methanol and double deionized water (ddH_2_O) with 0.1 % formic acid and flow rates of 0.5 mL/min or 0.8 mL/min depending on the respective method used (Additional file 1: Table S5).

Once resolved, compounds were detected in the UV/Vis spectrum at 280 nm first. Further, ionization was performed by Dual Ion Source, simultaneously combining electron spray ionization (ESI) and atmospheric pressure chemical ionization (APCI) for MS generation. Desolvation and block temperatures were kept at 275 and 400 °C, respectively. Nebulization gas flow was set at 1.5 L/min whereas the drying gas flow was set at 15 L/min. The ionized masses of the analytes were monitored in the positive ion mode between the mass-to-charge ratio (m/z) of 159–1000. The analytes were identified by their retention times (RT) and typical mass fragmentation patterns, either in comparison to commercially available authentic standards or literature references (Additional file 1: Table S6) [23, 25]. From measurements in both, MS and UV/Vis detection modes, the product/intermediate distribution was estimated setting all relevant peak areas except the substrate to 100 % (Additional file 1: Table S7). Concentrations of the final product (−)−deoxypodophyllotoxin **6** were calculated via an internal calibration using (+)-sesamin as IS (Additional file 1: Table S7). All values represent the average from biological and technical duplicates at least.

## Supplementary Information

**Additional file 1**: Supplementary materials include DNA-sequences of the genes used. Supplementary methods include detailed information about individual genes expression and reaction analytics. Supplementary results support the conclusions drawn in the main manuscript. **Table S1**. Summary of generated *cyp71cu1* and *cyp82d61* gene variants. **Table S2**. Summary of genes used in establishing of multi-enzyme cascade. **Table S3**. Summary of plasmids used within this study. **Table S4**. Oligonucleotides used for generation of *cyp71cu1* and *cyp82d61* gene variants. **Table S5**. LC/MS methods used within this study. **Table S6**. Qualitative metabolite analysis.**Table S7**. Quantitative metabolite analysis. **Table S8**. Expression levels of CYP71CU1  variants in *E. coli* C41(DE3) at 25°C after 48 h. **Table S9**. Expression levels of N-terminally truncated CYP71CU1 variants in *E. coli* C41(DE3) at various incubation temperatures after 48 h. **Table S10**. Expression levels of codon optimized WT and N-terminally truncated CYP82D61 variants in *E. coli* C41(DE3) at various incubation temperatures after 48 h. **Figure S1**. 12.5% SDS-gel of OMT3 (41 kDa; marked by red arrow) expression in various *E. coli* strains. **Figure S2**. 12.5% SDS-gel of OMT1 (38 kDa; marked by red arrow) expression in various *E. coli* strains. **Figure S3**. 12.5% SDS-gel of 2-ODD (35 kDa; marked by red arrow) expression in various *E. coli* strains. **Figure S4**. 12.5% SDS-gel of CYP71CU1 (56 kDa; marked by red arrow) expression in various *E. coli* strains. **Figure S5**. CO-difference spectra of soluble CYP71CU1OC_WT in *E. coli* BL21 (DE3) (orange line), C41 (DE3) (blue line), and C43 (DE3) (green line) after 24 h expression. **Figure S6**. CO-difference spectra of soluble CYP71CU1nat_WT (blue line) and CYP71CU1OC_WT (orange line) after 48 h expression. **Figure S7**. Expression analysis of CYP71CU1  variants incubated at different temperatures.** Figure S8**. LC/MS analysis (method 4) of one-cell biotransformations of (−)−matairesinol 1 to (−)−deoxypodophyllotoxin 6. **Figure S9**. LC/MS analysis (method 1) of OMT3 activity in the reaction cascade with CYP719A23. **Figure S10**. (A) CO-difference spectra of single expressed CYP71CU1∆20nat (orange line) and CYP71CU1∆20nat co-expressed with OMT3 (blue line). (B) 12.5% SDS-gel of CYP71CU1∆20nat (54 kDa) co-expression with OMT3 (41 kDa), and OMT1 (38 kDa) with 2-ODD (35 kDa). **Figure S11**. Schematic overview on the plasmid-based modular strategy developed to synthesize (−)−deoxypodophyllotoxin 6 from (−)−matairesinol 1 in *E. coli* via a one-cell 5-steps 6-enzyme reaction cascade. **Figure S12**. Hypothesized side reaction of 2-ODD occurring in the two-cell approach. **Figure S13**. Schematic overview on the plasmid-based modular strategy developed to synthesize (−)−epipodophyllotoxin 7 from (−)−matairesinol 1 in *E. coli* via a two-cell 6-steps 7-enzyme reaction cascade.

## Data Availability

All materials, as well as all raw data presented within this manuscript will be freely available to any scientist wishing to use them for non-commercial purposes, without breaching participant confidentiality.
