# Supplementary material for: Synthesis of (−)−deoxypodophyllotoxin and (−)−epipodophyllotoxin via a multi-enzyme cascade in E. coli
Source: Microb Cell Fact. 2021 Sep 20;20:183. doi: 10.1186/s12934-021-01673-5 (PMC8454061; doi:10.1186/s12934-021-01673-5)
Supplement: Supplementary file 1 — Additional file 1: Supplementary materials include DNA-sequences of the genes used. Supplementary methods include detailed information about individual genes expression and reaction analytics. Supplementary results support the conclusions drawn in the main manuscript. Table S1. Summary of generated cyp71cu1 and cyp82d61 gene variants. Table S2. Summary of genes used in establishing of multi-enzyme cascade. Table S3. Summary of plasmids used within this study. Table S4. Oligonucleotides used for generation of cyp71cu1 and cyp82d61 gene variants. Table S5. LC/MS methods used within this study. Table S6. Qualitative metabolite analysis.Table S7. Quantitative metabolite analysis. Table S8. Expression levels of CYP71CU1 variants in E. coli C41(DE3) at 25°C after 48 h. Table S9. Expression levels of N-terminally truncated CYP71CU1 variants in E. coli C41(DE3) at various incubation temperatures after 48 h. Table S10. Expression levels of codon optimized WT and N-terminally truncated CYP82D61 variants in E. coli C41(DE3) at various incubation temperatures after 48 h. Figure S1. 12.5% SDS-gel of OMT3 (41 kDa; marked by red arrow) expression in various E. coli strains. Figure S2. 12.5% SDS-gel of OMT1 (38 kDa; marked by red arrow) expression in various E. coli strains. Figure S3. 12.5% SDS-gel of 2-ODD (35 kDa; marked by red arrow) expression in various E. coli strains. Figure S4. 12.5% SDS-gel of CYP71CU1 (56 kDa; marked by red arrow) expression in various E. coli strains. Figure S5. CO-difference spectra of soluble CYP71CU1OC_WT in E. coli BL21 (DE3) (orange line), C41 (DE3) (blue line), and C43 (DE3) (green line) after 24 h expression. Figure S6. CO-difference spectra of soluble CYP71CU1nat_WT (blue line) and CYP71CU1OC_WT (orange line) after 48 h expression. Figure S7. Expression analysis of CYP71CU1 variants incubated at different temperatures. Figure S8. LC/MS analysis (method 4) of one-cell biotransformations of (−)−matairesinol 1 to (−)−deoxypodophyllotoxin 6. Figure [file 12934_2021_1673_MOESM1_ESM.pdf]

Synthesis of (-)-deoxypodophyllotoxin and (-)-epipodophyllotoxin via a multi-enzyme cascade in *E. coli*

Davide Decembrino, Alessandra Raffaele, Ronja Knöfel, Marco Girhard and Vlada B. Urlacher*

Institute of Biochemistry, Heinrich-Heine University Düsseldorf, Universitätsstraße 1, 40225 Düsseldorf, Germany

* Corresponding author
vlada.urlacher@uni-duesseldorf.de

Supplementary Information

1. **Supplementary Material 2**
2. **Supplementary Methods 5**
3. **Supplementary Results 10**
4. **Supplementary References 22**

1. Supplementary Material

omt1 (GenBank KT390155.1), native DNA-sequence

ATGGATACTAGGGCTGATGCTGAGATTAAAGCAATGGAGCTGATTGGTATTGGAGTACTTCCACTGGCAATGA
AGGCGATAATTGAGCTCAATGTGTTAGAGATCCTATCAAAAGCAGGACCAGATACCCAACTCACTGCTGCTCA
AATTGTCACCGACATACCCACCACCAACCCTAACGCTGGTTTCCAACTAGATCGAATTTTACGACTACTAGCA
AGTCATTCAGTCTTGTCTAGTAGTATTACAAAATCGGGGGAGAGAGTGTATGGGCTAACCCCTATGTGCAAAT
ACTTTCTCCCAGATCAAGATGGAGTCTCACTAGCACCTATGGTTGTTACCATCCATGACAAAGTGCTGCTTCA
AAGTTGGCATTATCTTAAGGACTCTGTTTTAAAACAAGGCTCTTTGCCATTTACCGAGGCCTTTGGGATGTCA
CCCTTTGAGTATTCTGTCTCCGATACAAGGTTTAATAAGGTTTTTAATGCTGGCATGTTTGACCATTCTACTC
TTTGTATGAGGGATGTCCTTCAACGGTACAAAGGATTTCAAGGCTTGGGGGAGCTGGTTGATGTTGGTGGAGG
AACTGGCGGATCGTTGAAGATGATTCTTTCTCAGTACCCCAATCTAAAGGGCATCAATTTTGATCTCCCACAT
GTGGTTGCCGACGCGCCTTCTTTTCCTGGTGTGAAGCACATTGGTGGTGATATGTTTGAGAGTGTTCCCTCTG
GTGATGCAATTTTCATGAAGTGGATACTTCATGACTGGGACGATGGACGTTGCCTGACTTTGCTGAAGAATTG
TTGGAATGCATTGCCAGAGCATGGAAAGGTGATAATAGTGGAGTGGATTCTACCATCAGATGCAGCGACTGAC
CCAACATCTCGCCGTGTCTTCACAGCTGATTTGATGATGTTGGCTTTCAGCGAAGGGGGTAAAGAGCGAACCT
TGGGTGACTACGGAGCACTTGCAAAGGAAGCTGGTTTTACCACTGTCAAAGATTTCCCTTGCGCAAATGGCAT
TTCAGTCATTGAGTTCCATAAGAAGTGA

omt3 (GenBank KT390157.1), native DNA-sequence

ATGGAAATGGCTCCAACAATGGATTTAGAGATAAGAAATGGAAATGGTTATGGTGATTCTGGAGAGGAGCTTC
TAGCAGCACAAGCTCACATATACAACCACATATTCAACTTCATAAGCTCGATGGCACTGAAATGTGCAGTGGA
GTTAAACATACCAGAAATTCTCCACAACCATCAACCCAAAGCGGTTACTCTCTCTGAACTAGTACAGGCCCTT
CAAATCCCCCAAGCAAAATCCGCGTGTCTGTATCGCCTGTTGCGAATACTAGTCCATTCTGGCTTCTTTGCCA
TAACGAAAATACAAAGCGAGGGAGATGAAGAGGGTTATTTACCAACCCTTTCCTCTAAACTACTACTGAAAAA
CCATCCCATGAGCATGTCTCCATGCTTGTTAGGACTGGTGAATCCTACAATGGTAGCACCCATGCATTTCTTT
AGTGATTGGTTCAAGAGGAGTGATGATATGACGCCGTTTGAGGCGACGCATGGAGCGAGCTTGTGGAAGTATT
TTGGTGAAACCCCACACATGGCGGAGATATTTAATGAGGCAATGGGTTGTGAGACAAGGTTGGCGATGAGTGT
GGTGTTGAAAGAGTGTAAGGGCAAGCTTGAAGGAATAAGTTCGTTAGTTGATGTAGGAGGTGGTACAGGAAAC
GTGGGTCGGGCAATTGCTGAAGCCTTCCCAAATGTCAAGTGCACCGTGTTAGATTTGCCACAAGTTGTTGGAA
ACTTGAAAGGCAGTAACAATTTGGAGTTTGTCAGTGGGGATATGTTTCAATTTATTCCGCCTGCAGACGTAGT
TTTCTTGAAGTGGATATTGCATGATTGGAATGATGAGGAATGTATAAAAATCCTAAAGAGGTGCAAGGAAGCG
ATTCCATCCAAGGAAGAGGGAGGGAAATTGATCATAATAGACATGGTAGTAAACGACCACAACAAGGGAAGCT
ATGAGTCTACAGAAACGCAACTCTTCTATGATTTGACGCTCATGGCTCTGTTGACAGGAACAGAGAGAACCGA
AACTGAATGGAAGAAGCTCTTCGTAGCTGCTGGTTTCACAAGTTACATTATTAGCCCTGTTTTGGGGCTCAAG
TCTATCATTGAAGTGTTTCCCTAA

***cyp71cu1* (GenBank KT390172.1), native DNA-sequence (*cyp71cu1nat_wt*)**

N-terminally truncated part of the DNA-sequence (*CYP71CU1Δ20nat*) is highlighted in bold and underlined

ATG**GAAACATTTCAGTGCCTCACACTTTTCCTTCTCTTCATCTCCACTGTTTTCATCCTCAAG**AGAAAATTCT
CTCATAAACCAAACCTACCACCATCACCACCAAAGCTACCAATCCTAGGCAACTTTCATCAGCTAGGCACACT
TGTCCACCGAGCTGTTACAGTTCTCGCAGCCAAATATGGCCCCCTCATGCTCTTACACTTTGGCAAAACTCCA
GTTCTCATTGTTTCCTCACAAGAAACGGCTAAAGAGATCATGAAAACCCATGACCTCGCTTTGGCAAATAGAC
CCCTAACAACTGCTGCCAGGGCACTACTTTACGACTGCACAGATATATCTTTTGCACCTTATGGTGAGTACTG
GAGAGAAATGAAAAAGATGGCAGTCCTAAACCTTCTTAGTATCAAAAAAATCCAGTCGTTTCGATCCGTTAGG
GAGGAATTGGCTTCTGATATGATAAAGGAGATTACTCGTTTGTCCAAAACTGGAGCACCTGTGGATGTAACTA
ATATGTTATATCATTTTTCCGAAGACCTGCTTTTTAGGTGTACTCTTGGGTTTAAACCTAAGGGACAACACAA
GTTTCAGAAGCTGTCCAGGGATTTTTTGGATCTTGTAGGAGCTTTTTGTTTTAATGATTTCTTCCCAGGGATG
GCATGGATGGATGCTCTCACTGGGCTAAACAGGAAGCTGAAGAAGGGTTCAAGAGAATTAGATGACTTTGTTG
ATGAACTCATAGAAGAACGCATTGCTATGGTAAAAGATGGCGTTGAGCCAAATGAATTTCTGGATCTTCTACT
CCATACTCATAGAGACACCACCCAGGAAATCAAACTCACCCGAGACAACGTCAAAGCAATAATATTGGACACA
TTTCTTGGTGGAATTGATCTACCAGCATCAGTCATGGAATGGGCAATGGCCGAGCTTATGAGGAATCCAAGTA
AGATGAAGATAGCTCAGGAAGAGGTCAGAAAAGTGGTTGGAAACAAAAACAAGGTAGACGAAGACGATGTGTA
TCAAATGAATTTCTTGAAATCGGCTGTTAAGGAAACTCTAAGGCTACACCCACCAGCTCCTTTGCTATTTGCG
AGAGAGTCATATACAAGTATAAACGTCGAGAACTACATCATTCCTCCTTACACTAGTGTCATGATCAACATCT
GGCATATCCAAAGGGACCCCAAGTTATGGGACAAGGCCGAAGAGTTTATTCCAGAGAGATTCATGAACAGCGG
GATTGATTACAAATCCCATGACTATGAATTCATCCCTTTTGGATCCGGACGAAGAGGTTGCCCTGGTATGTCG
TTTGGCGTTGCAGCAGTGGAGTTTGCTGTTGCCAATCTCTTATACTGGTTTGATTGGAAGTTTGTTGGTGATA
CAACTCCTGAAACACTTGATATGACTGAGGACTATTGTTTTGCCCTCTTTAAGAAAAAACCTTTGCATTTTAT
TCCTATTTCTCGATCCTCTTGA

***cyp71cu1*, *E. coli* codon optimized DNA-sequence (c*yp71cu1oc_wt*)**

N-terminally truncated part of the DNA-sequence (*cyp71cu1Δ20oc*) is highlighted in bold and underlined

ATG**GAAACCTTCCAGTGCCTGACCCTGTTCCTGCTGTTCATCTCTACCGTTTTCATCCTGAAA**CGTAAATTCT
CTCACAAACCGAACCTGCCGCCGTCTCCGCCGAAACTGCCGATCCTGGGTAACTTCCACCAGCTGGGTACCCT
GGTTCACCGTGCTGTTACCGTTCTGGCTGCTAAATACGGTCCGCTGATGCTGCTGCACTTCGGTAAAACCCCG
GTTCTGATCGTTTCTTCTCAGGAAACCGCTAAAGAAATCATGAAAACCCACGACCTGGCTCTGGCTAACCGTC
CGCTGACCACCGCTGCTCGTGCTCTGCTGTACGACTGCACCGACATCTCTTTCGCTCCGTACGGTGAATACTG
GCGTGAAATGAAAAAAATGGCTGTTCTGAACCTGCTGTCTATCAAAAAAATCCAGTCTTTCCGTTCTGTTCGT
GAAGAACTGGCTTCTGACATGATCAAAGAAATCACCCGTCTGTCTAAAACCGGTGCTCCGGTTGACGTTACCA
ACATGCTGTACCACTTCTCTGAAGACCTGCTGTTCCGTTGCACCCTGGGTTTCAAACCGAAAGGTCAGCACAA
ATTCCAGAAACTGTCTCGTGACTTCCTGGACCTGGTTGGTGCTTTCTGCTTCAACGACTTCTTCCCGGGTATC
TTGGATGGACGCTCTGACCGGTCTGAACCGTAAACTGAAAAAAGGTTCTCGTGAACTGGACGACTTCGTTGAC
GAACTGATCGAAGAACGTATCGCTATGGTTAAAGACGGTGTTGAACCGAACGAATTTCTGGACCTGCTGCTGC
ACACCCACCGTGACACCACCCAGGAAATCAAACTGACCCGTGACAACGTTAAAGCTATCATCCTGGACACCTT
CCTGGGTGGTATCGACCTGCCGGCTTCTGTTATGGAATGGGCTATGGCTGAACTGATGCGTAACCCGTCTAAA
ATGAAAATCGCTCAGGAAGAAGTTCGTAAAGTTGTTGGTAACAAAAACAAAGTTGACGAAGACGACGTTTACC
AGATGAACTTCCTGAAATCTGCTGTTAAAGAAACCCTGCGTCTGCACCCGCCGGCTCCGCTGCTGTTCGCTCG
TGAATCTTACACCTCTATCAACGTTGAAAACTACATCATCCCGCCGTACACCTCTGTTATGATCAACATCTGG
CACATCCAGCGTGACCCGAAACTGTGGGACAAAGCTGAAGAATTTATCCCGGAACGTTTCATGAACTCTGGTA
TCGACTACAAATCTCACGACTACGAATTTATCCCGTTCGGTTCTGGTCGTCGTGGTTGCCCGGGTATGTCTTT
CGGTGTTGCTGCTGTTGAATTTGCTGTTGCTAACCTGCTGTACTGGTTCGACTGGAAATTCGTTGGTGACACC
ACCCCGGAAACCCTGGACATGACCGAAGACTACTGCTTCGCTCTGTTCAAAAAAAAACCGCTGCACTTCATCC
CGATCTCTCGTTCTTCTTAA

2-odd (GenBank KT390173.12), native DNA-sequence

ATGGGTTCTACAGCACCCCTAAGGCTTCCAGTTATAGATTTATCCATGAAGAACTTGAAGCCTGGAACAACTT
CTTGGAACTCGGTACGCACCCAGGTACGGGAGGCACTGGAAGAATACGGTTGCTTTGAAGCTGTGATCGATGC
TGTGTCTCCAGAGCTGCAGAAGGCAGTATGTAACAAAGGACACGAGCTGCTTAATCTTCCATTAGAAACCAAG
ATGTTGAACGGAAACAAACCAGAATATGATGGATTTACGTCAATACCAAACCTCAACGAAGGCATGGGAGTCG
GCAGAATAACAGATTTGGAAAAAGTTGAGAGGTTCACTAATCTTATGTGGCCCGAGGGGAATAAGGATTTCTG
TGAAACTGTGTATTCTTATGGCAAACGAATGGCGGAGGTGGACCACATATTGAAAATGATGGTTTTCGAGAGT
TTTGGAATGGAGAAGCACTTCGACTCGTTCTGCGAATCAACAAATTACCTTCTCCATTTCATGAGATACCAAC
AACCAGGGAAGGATGGACGTTCACCTGCTCTTTCGTTGCATAAGGACAAGAGCATCTTGACCATAGTAAACCA
AAATGATGTCAAGGGATTGGAATTTGAAACCAAGGATGGAGAATGGATTTTACCTACAGCTGACAACCATATT
GTTCTTCTAGGAGACTGCTTCATGGCATGGAGCAATGGTAGATTACATAGTCCTCTTCACCGGGTCACGTTGG
TCGCGAACCAGGCGAGGTTATCTACATCATCGTTTTCGTTTCCAAAGGACATAATAGAGACCCCTGCAGAGCT
GGTGGATGAAGAGCATCCTTTGCTATTTAATCCCTTTGAGATAACGGAGTTGCTTGCTTACTGTTTCACAAAA
GAGGGTGCAAAGGCGGTGTGTGACCTCAAGCAATACAAGGCGTACACAGGTGCATGA

cyp82d61, *E. coli* codon optimized DNA-sequence (*cyp82d61oc_wt*)

N-terminally truncated part of the DNA-sequence (*cyp82d61Δ23oc*) is highlighted in bold and underlined; for native DNA-sequence of *cyp82d61* gene see GenBank KC110995.1

ATG**GACTCTCTGCACTGCCTGGAAACCCTGCTGCTGGGTTTCTTCGTTCTGCTGCCGTGCTTCTTCTACTTC**G
TTTGGAAAAAACCGAACAACAAAATCAAAGAACCGCCGCAGCCGGCTGGTGCTTGGCCGATCATCGGTCACCT
GCACCTGCTGGCTCGTGGTGACCTGCCGCACAAAATCCTGTCTTCTTTCGCTGACAAAAACGGTCCGGTTTTC
AAAATCCAGCTGGGTGTTCACCAGGCTCTGGTTGTTAACAACTCTGAAATCGCTAAAGAATGCTTCACCACCA
ACGACCGTTTCTTCCTGAACCGTCCGTCTGGTGTTGCTGCTAAAATCATGGGTTACAACTACGTTATGCTGGG
TGTTGCTCCGTACGGTCCGTACTGGCGTGACATGCGTAAAATCATCATGCTGGAATTCCTGTCTAACCGTCGT
CTGCAGTCTCTGAAACACGTTTGGCACTCTGAAATCTCTATCTCTTCTAAAGAACTGTACAAACTGTGGGAAA
CCCAGAACATCGACTTCTGCCTGGTTGACATGAAACAGTGGCTGGCTGACCTGACCCTGAACATGTCTGTTAA
AATGGTTGTTGGTAAACGTTTCTTCGGTTCTGCTTCTGCTTCTGCTTGCGAAGAAACCGAATCTTCTAACTGC
CCGAAAACCCTGCGTAACATGTTCCGTCTGATGGGTTCTTTCGTTCTGTCTGACTACCTGCCGTACCTGCGTT
GGCTGGACCTGGGTGGTCACGAAAAAGAAATGAAACGTACCGTTAAAGAACTGGACATCCTGTTCAAAGGTTG
GCTGGACGAACACAAACGTAAACGTCTGTCTGGTGGTAAAGAAGACGACGACCAGGACTTCATGGACGTTATG
CTGTCTATCCTGGAAGAATCTAAACTGGGTAACGACGTTGACACCATCAACAAAACCGCTTGCCTGGCTATCA
TCCTGGGTGGTGCTGACACCACCTGGGCTACCCTGACCTGGGCTCTGTCTCTGCTGCTGAACAACCCGAACGC
TCTGAAAAAAGCTCAGGACGAACTGGACCTGCACGTTGGTCGTGACCGTAACGTTGACGAATCTGACCTGGTT
AAACTGACCTACATCGACGCTATCATCAAAGAAACCCTGCGTCTGTACCCGCCGGGTCCGCTGCTGGGTCCGC
GTGTTGTTACCGAAGACTGCACCATCGCTGGTTACCACGTTCGTGCTGGTACCCGTCTGATCGTTAACGCTTG
GAAAATCCAGCGTGACCCGCTGGTTTGGTCTCAGCCGCACGAATACCAGCCGGAACGTTTCCTGGAACGTGAC
GTTGACATGAAAGGTCAGCACTTCGAACTGATCCCGTTCGGTTCTGGTCGTCGTGCTTGCCCGGCTATCTCTC
TGGCTCTGCAGGTTCTGCCGCTGACCCTGGCTCACATCCTGCACGGTTTCGAACTGCGTACCCCGAACCAGAA
CAAAGTTGACATGACCGAAACCCCGGGTATCGTTCACGCTAAAGCTACCCCGCTGGAAGTTCTGGTTGCTCCG
CGTATCTCTCCGAAATGCTTCGTTTAA

2. Supplementary Methods

Expressions of OMT3, OMT1 and 2-ODD

Expressions were evaluated in *E. coli* BL21 (DE3), C41(DE3) and C43 (DE3). Protein expression was induced at OD_{600} = 0.6 by the addition of 0.5 mM IPTG (additional supplementation with 0.1 mM $FeSO_4$ in the case of 2-ODD), followed by incubation at 25°C, 120 rpm for 20 h.

Expressions of wildtype and N-terminal truncated CYP71CU1 and CYP82D61 variants

Expressions were evaluated in *E. coli* C41(DE3). Protein expression was induced at OD_{600} = 0.6 by the addition of 0.5 mM IPTG, with additional supplementation with 0.5 mM 5-aminolevulinic acid (5-ALA) and 0.1 mM $FeSO_4$. Incubations were carried out at 120 rpm for 20 h or 48 h, with temperatures of 20°C, 25°C and 30°C. Cells were harvested by centrifugation (3,220 x g, 4°C, 20 min) and the cell pellet was resuspended in 50 mM potassium phosphate buffer, pH 7.5, 100 µM PMSF. Cells were disrupted by sonication, the cell debris was sedimented by centrifugation (12,300 x g, 4°C ,30 min) and separated from the soluble protein fraction.

P450 concentrations within the soluble protein fractions were determined by recording CO difference spectra according to the method of Omura and Sato [1] to allow comparison between wildtype and truncated versions of CYP71CU1 and CYP82D61.

The results of the screening and optimized expression conditions are summarized in Tables S8, S9, and S10 within this appendix.

Table S1. Summary of generated *cyp71cu1 and cyp82d61* gene variants.

Name of variant	DNA-sequence	Inserted modification (second amino acid)	No. of N-terminal truncated amino acids
cyp71cu1Δ20nat	Native	Ala	1 - 20 (Δ20)
cyp71cu1Δ20oc	*E. coli* codon optimized	Ala	1 - 20 (Δ20)
cyp82d61Δ23oc	*E. coli* codon optimized	Ala	1 - 23 (Δ23)

Table S2. Summary of genes used for establishment of multi-enzyme cascade reactions.

Gene	Complete name or purpose	Organism	Reference
cyp719aΔ23oc	(-)-pluviatolide synthase (truncated, codon optimized)	*Sinopodophyllum hexandrum*	Decembrino *et al.*, 2020 [2]
atr2	NADPH-cytochrome P450 reductase 2 (ATR2)	*Arabidopsis thaliana*	Kranz-Finger *et al.*, 2018 [3]
omt3	(-)-pluviatolide-O-methyltransferase	*Sinopodophyllum hexandrum*	This work
cyp71cu1Δ20nat	(-)-5'-desmetoxy-yatein hydroxylase (truncated, native DNA-sequence)	*Sinopodophyllum hexandrum*	This work
omt1	(-)-5'-desmethyl-yatein O-methyltransferase	*Sinopodophyllum hexandrum*	This work
2-odd	(-)-deoxypodophyllotoxin synthase	*Sinopodophyllum hexandrum*	This work
cyp82d61Δ23oc	Putative hydroxylation (truncated, codon optimized)	*Sinopodophyllum hexandrum*	This work

Table S3. Summary of plasmids used within this study.

Plasmid	Features	Copy number	Reference
pETDuet_atr2_cyp719aΔ23oc	ColE1 ori, P_{T7}, *lacI,* Amp^R	~ 40	Decembrino *et al.,* 2020 [2]
pCDFDuet_omt3_cyp71cu1Δ20nat	CloDf13 ori, P_{T7}, *lac,* Sm^R	20 - 40	This work
pCDFDuet_omt3	CloDf13 ori, P_{T7}, *lac,* Sm^R	20 - 40	This work
pCDFDuet_cyp71cu1Δ20nat	CloDf13 ori, P_{T7}, *lac,* Sm^R	20 - 40	This work
pCDFDuet_cyp71cu1Δ20oc	CloDf13 ori, P_{T7}, *lac,* Sm^R	20 - 40	This work
pCDFDuet_cyp71cu1nat_wt	CloDf13 ori, P_{T7}, *lac,* Sm^R	20 - 40	This work
pCDFDuet_cyp71cu1oc_wt	CloDf13 ori, P_{T7}, *lac,* Sm^R	20 - 40	This work
pCOLADuet_2-odd_omt1	ColA ori, P_{T7}, *lac,* Kan^R	20 - 40	This work
pCDFDuet_omt1	CloDf13 ori, P_{T7}, *lac,* Sm^R	20 - 40	This work
pCDFDuet_2-odd	CloDf13 ori, P_{T7}, *lac,* Sm^R	20 - 40	This work
pETDuet_atr2_cyp82d61Δ23oc	ColE1 ori, P_{T7}, *lacI,* Amp^R	~ 40	This work
pETDuet_cyp82d61oc_wt	ColE1 ori, P_{T7}, *lacI,* Amp^R	~ 40	This work
pETDuet_cyp82d61Δ23oc	ColE1 ori, P_{T7}, *lacI,* Amp^R	~ 40	This work

Table S4. Oligonucleotides used for generation of *cyp71cu1 and cyp82d61* gene variants.

Name of variant	Oligonucleotides 5' → 3'
cyp71cu1Δ20nat	**forward:** CAGAAAATTCTCTCATAAACCAAAC<br>**reverse:** GCCATATGTATATCTCCTTCTTTATAC
cyp71cu1Δ20oc	**forward:** CCGTAAATTCTCTCACAAAC<br>**reverse:** GCCATATGTATATCTCCTTCTTATAC
cyp82d61Δ23oc	**forward:** CGTTTGGAAAAAACCGAAC<br>**reverse:** GCCATATGTATATCTCCTTCTTATAC

Table S5. LC/MS methods used within this study. Gradients were made of methanol (solvent A) and ddH_2O + 0.1% formic acid (solvent B). A flow rate of 0.5 mL/min was employed with methods 1, 2 and 3, whereas 0.8 mL/min was used in case of method 4.

	**Time [min]**	**Solvent B [%]**
	0.01	57
**Method 1**	8.00	28
	13.00	0
	14.01	57
	20.00	57

	**Time [min]**	**Solvent B [%]**
	0.01	80
	5.00	65
**Method 2:**	10.00	65
	20.00	38
	25.00	0
	26.01	80
	35.00	80

	**Time [min]**	**Solvent B [%]**
	0.01	65
	5.00	50
**Method 3**	8.00	45
	22.00	38
	25.00	0
	26.01	80
	35.00	80

	**Time [min]**	**Solvent B [%]**
	0.01	80
	2.00	60
**Method 4**	5.00	55
	25.00	52
	32.50	0
	35.01	80
	40.00	80

Qualitative analysis

Table S6. Authentic reference compounds used for LC/MS identification of reaction products. Commercially not available compounds were identified via their characteristic m/z fragments as described elsewhere [4].

Compound	Manufacturer or reference	Purity	Molecular weight	RT [min] (method 4)	Characteristic m/z fragments
**(-)-matairesinol 1**	Phytolab	≥99% (HPLC)	358.38	9.0	359 $[M+H]^+$<br>341 $[M+H-H_2O]^+$
**(-)-pluviatolide 2**	*Decembrino et al. 2020* (isolated in-house) [2]	≥95% (HPLC)	360.61	15.0	357 $[M+H]^+$<br>339 $[M+H-H_2O]^+$
**(-)-yatein 5**	BioBioPha	≥97% (HPLC)	400.40	20.6	401 $[M+H]^+$<br>383 $[M+H-H_2O]^+$
**(-)-deoxypodophyllotoxin 6**	Toronto Research Chemical	≥98% (HPLC)	398.40	19.8	399 $[M+H]^+$<br>421 $[M+Na]^+$
**(-)-5'-desmethoxy-yatein 3**	Commercially not available [4]	-	370.40	21.0	$\underline{371\ [M+H^+]^+}$<br>$\underline{353\ [M+H-H_2O]^+}$
**(-)-5'-desmethyl-yatein 4**	Commercially not available [4]	-	386.40	14.8	$\underline{387\ [M+H]^+}$<br>$\underline{369\ [M+H-H_2O]^+}$
**(-)-epipodophyllotoxin 7**	Commercially not available [4]	-	414.41	10.4	$\underline{415\ [M+H]^+}$<br>437 $[M+Na]^+$
**(+)-sesamin (internal standard; IS)**	TCI	>98% (GC)	354.35	31.4	337 $[M+H-H_2O]^+$

Underlined m/z fragments correspond to those referred by Lau and Sattely [4].

Quantitative analysis

Table S7. For quantitative analysis, substrate conversion values were calculated by the product distribution resulting from the MS-peak areas of reaction intermediates/products and substrate. Estimation of the concentration of the final product (-)-deoxypodophyllotoxin **6** was done via internal calibration in the range of 10 - 300 µM utilizing 200 µM (+)-sesamin as IS.

**Conversion [%]** Normalized to IS	$= 1- (S_{sample} / IS_{sample}) / (S_{control} / IS_{control})$
**Conversion [%]**	$= \Sigma(P_{area}) / \Sigma(S_{area} + P_{area}) * 100$
**Product distribution [%]**	$= P_{area} / \Sigma(S_{area} + P_{area}) * 100$

IS: internal standard; S: substrate; P = product.

3. Supplementary Results

Expressions of OMT3, OMT1, 2-ODD and CYP71CU1

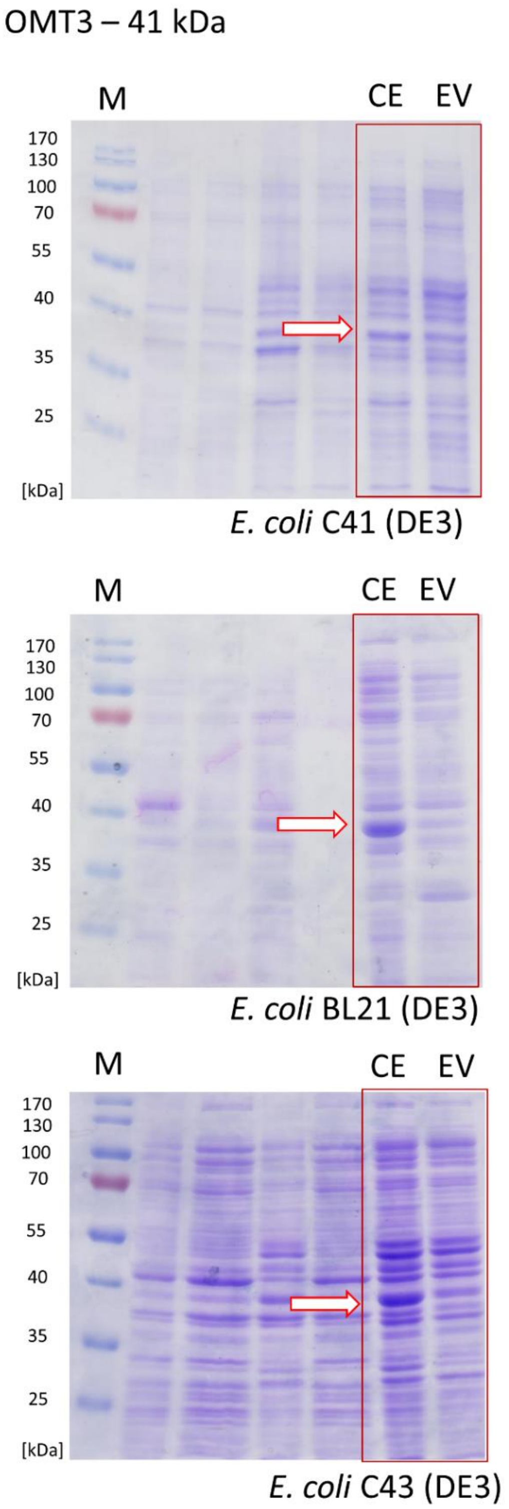

Figure S1. 12.5% SDS-gel of OMT3 (41 kDa; marked by red arrow) expression in various *E. coli* strains. M: Molecular weight size marker, EV: Vector control without *omt3* gene, CE: Soluble protein fraction after cell disruption.

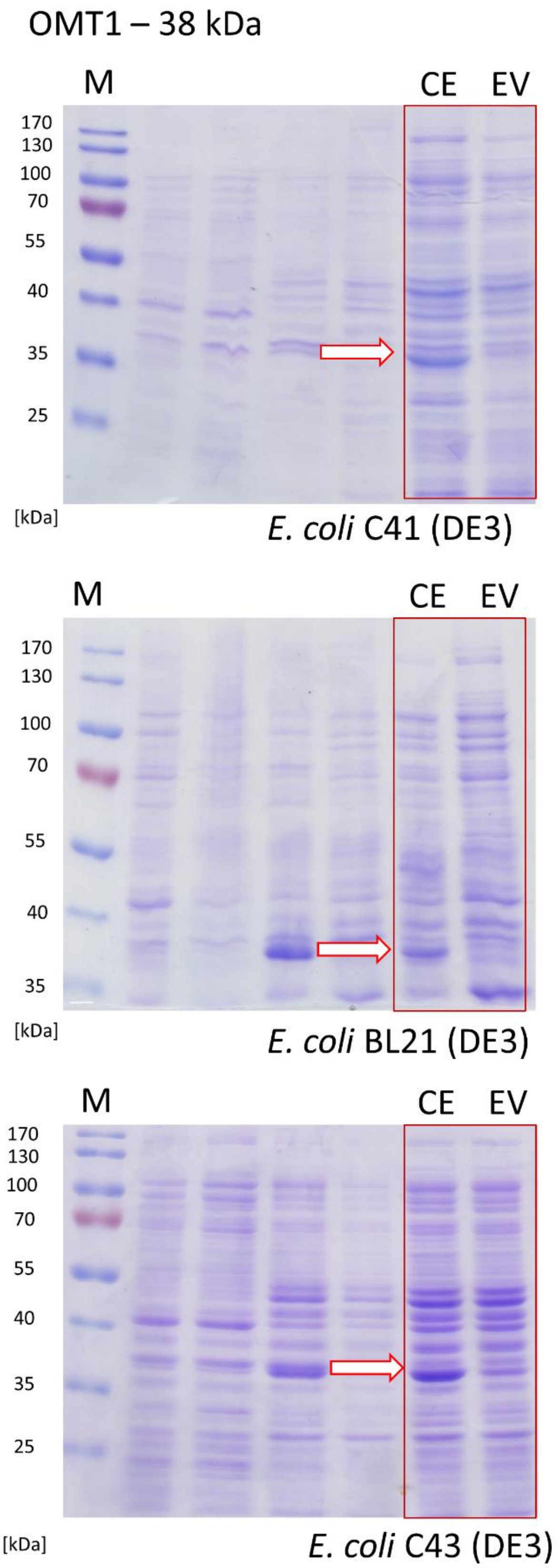

Figure S2. 12.5% SDS-gel of OMT1 (38 kDa; marked by red arrow) expression in various *E. coli* strains. M: Molecular weight size marker, EV: Vector control without *omt1* gene, CE: Soluble protein fraction after cell disruption.

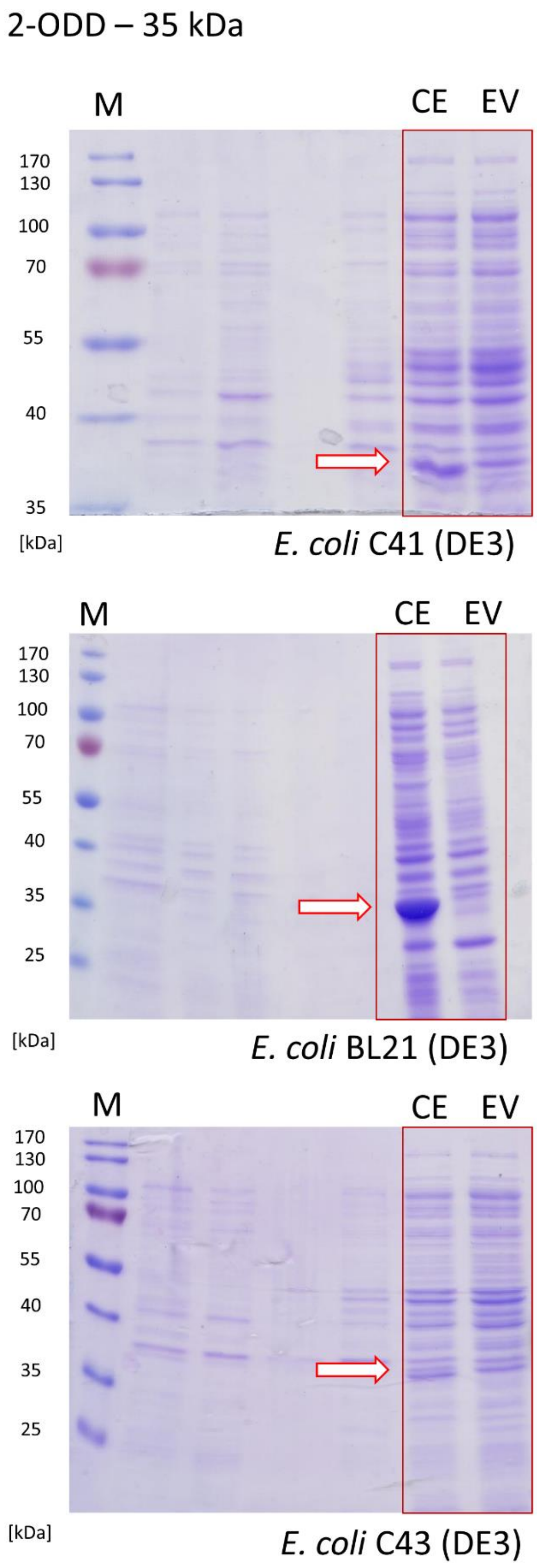

Figure S3. 12.5% SDS-gel of 2-ODD (35 kDa; marked by red arrow) expression in various *E. coli* strains. M: Molecular weight size marker, EV: Vector control without *2-odd* gene, CE: Soluble protein fraction after cell disruption.

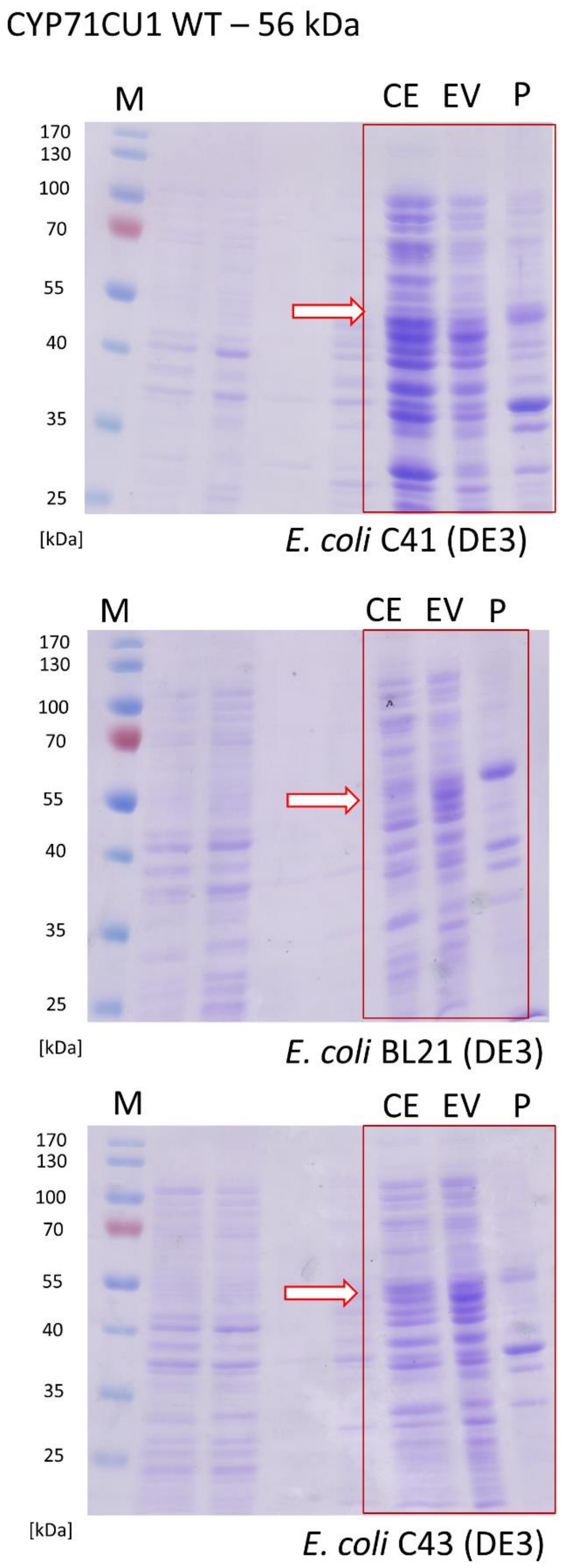

Figure S4. 12.5% SDS-gel of CYP71CU1 (56 kDa; marked by red arrow) expression in various *E. coli* strains. M: Molecular weight size marker, EV: Vector control without *cyp71cu1* gene, CE: Soluble protein fraction after cell disruption, P: Insoluble protein fraction after cell disruption.

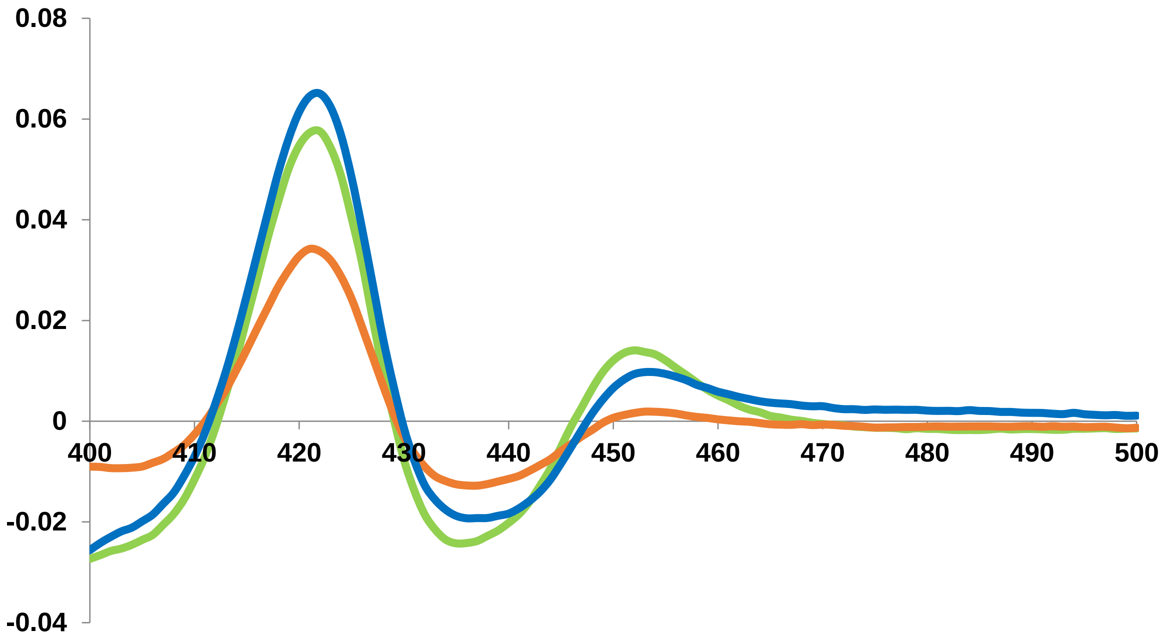

Figure S5. CO-difference spectra of soluble CYP71CU1OC_WT in *E. coli* BL21 (DE3) (orange line), C41 (DE3) (blue line), and C43 (DE3) (green line) after 24 h expression.

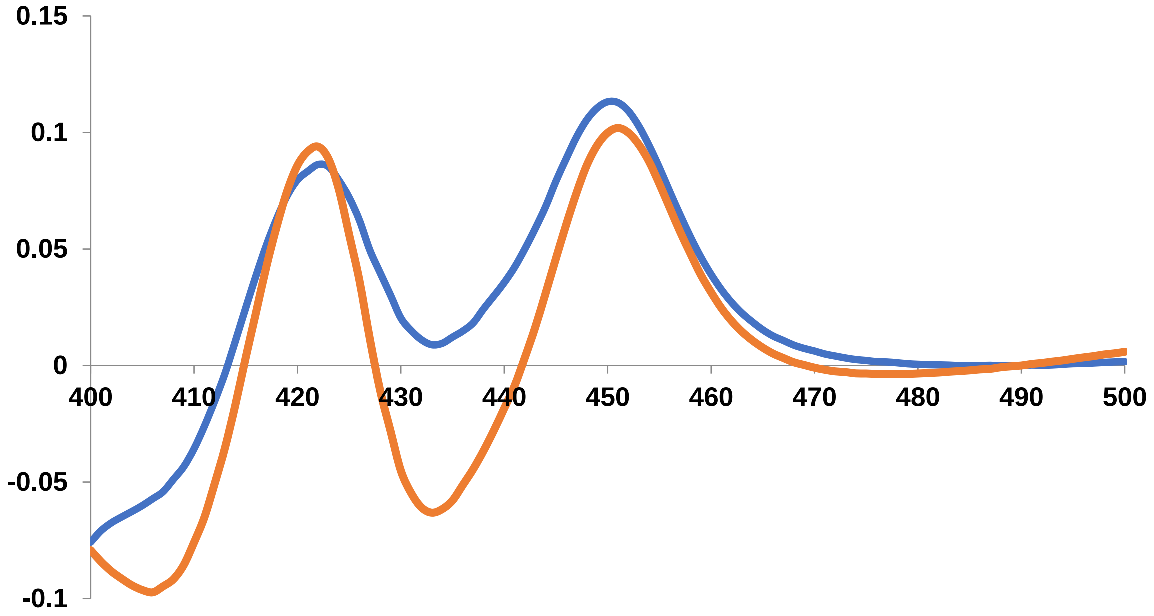

Figure S6. CO-difference spectra of soluble CYP71CU1nat_WT (blue line) and CYP71CU1OC_WT (orange line) after 48 h expression.

Table S8. Expression levels of CYP71CU1 variants in *E. coli* C41(DE3) at 25°C after 48 h.

Variant	Inserted modification	Truncated amino acids	P450 concentration	
			[µg/g_{cww}]	[mg/$l_{culture\ volume}$]
**CYP71CU1nat_WT**	-	-	263 ± 16	8 ± 5
**CYP71CU1Δ20nat**	Ala	1 - 20	813 ± 163	26 ± 6
**CYP71CU1oc_WT**	-	-	159 ± 16	6 ± 1
**CYP71CU1Δ20oc**	Ala	1 - 20	465 ± 138	15 ± 4

Table S9. Expression levels of N-terminally truncated CYP71CU1 variants in *E. coli* C41(DE3) at various incubation temperatures after 48 h.

Temperature	P450 concentration			
	[µg/g_{cww}]		[mg/$l_{culture\ volume}$]	
	CYP71CU1Δ20oc	CYP71CU1Δ20nat	CYP71CU1Δ20oc	CYP71CU1Δ20nat
**20°C**	282 ± 26	566 ± 39	11 ± 1	20 ± 3
**25°C**	465 ± 138	813 ± 163	15 ± 4	26 ± 6
**30°C**	112 ± 18	253 ± 108	6 ± 3	8 ± 3

Table S10. Expression levels of N-terminally truncated CYP82D61 variants in *E. coli* C41(DE3) at various incubation temperatures after 48 h.

Temperature	P450 concentration			
	[µg/g_{cww}]		[mg/$l_{culture\ volume}$]	
	CYP82D61oc_WT	CYP82D61Δ23oc	CYP82D61oc_WT	CYP82D61Δ23oc
**20°C**	n.d.	20 ± 17	n.d.	0.7 ± 0.6
**25°C**	n.d.	936 ± 34	n.d.	33 ± 2
**30°C**	250 ± 14	1395 ± 220	8 ± 1	42 ± 9

n.d.: Expression not detectable

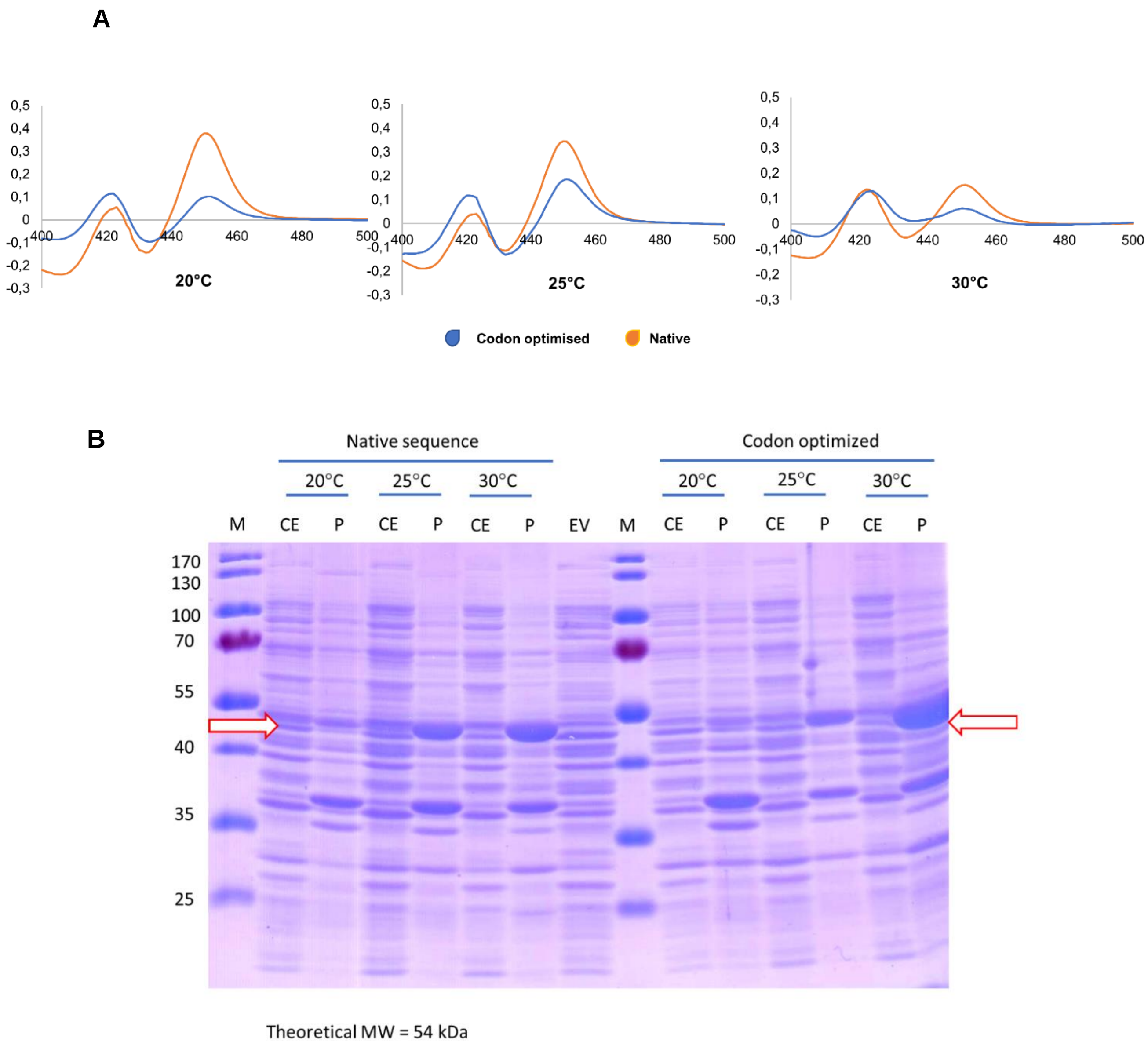

Figure S7. Expression analysis of CYP71CU1 variants incubated at different temperatures. **(A)** CO-difference spectra of CYP71CU1Δ20nat (orange line) and CYP71CU1Δ20oc (blue line). **(B)** 12.5% SDS-gel of CYP71CU1Δ20nat and CYP71CU1Δ20oc expressions (54 kDa; marked with a red arrow). M: Molecular weight size marker, EV: Vector control without *cyp71cu1* gene, CE: Soluble protein fraction after cell disruption, P: Insoluble protein fraction after cell disruption.

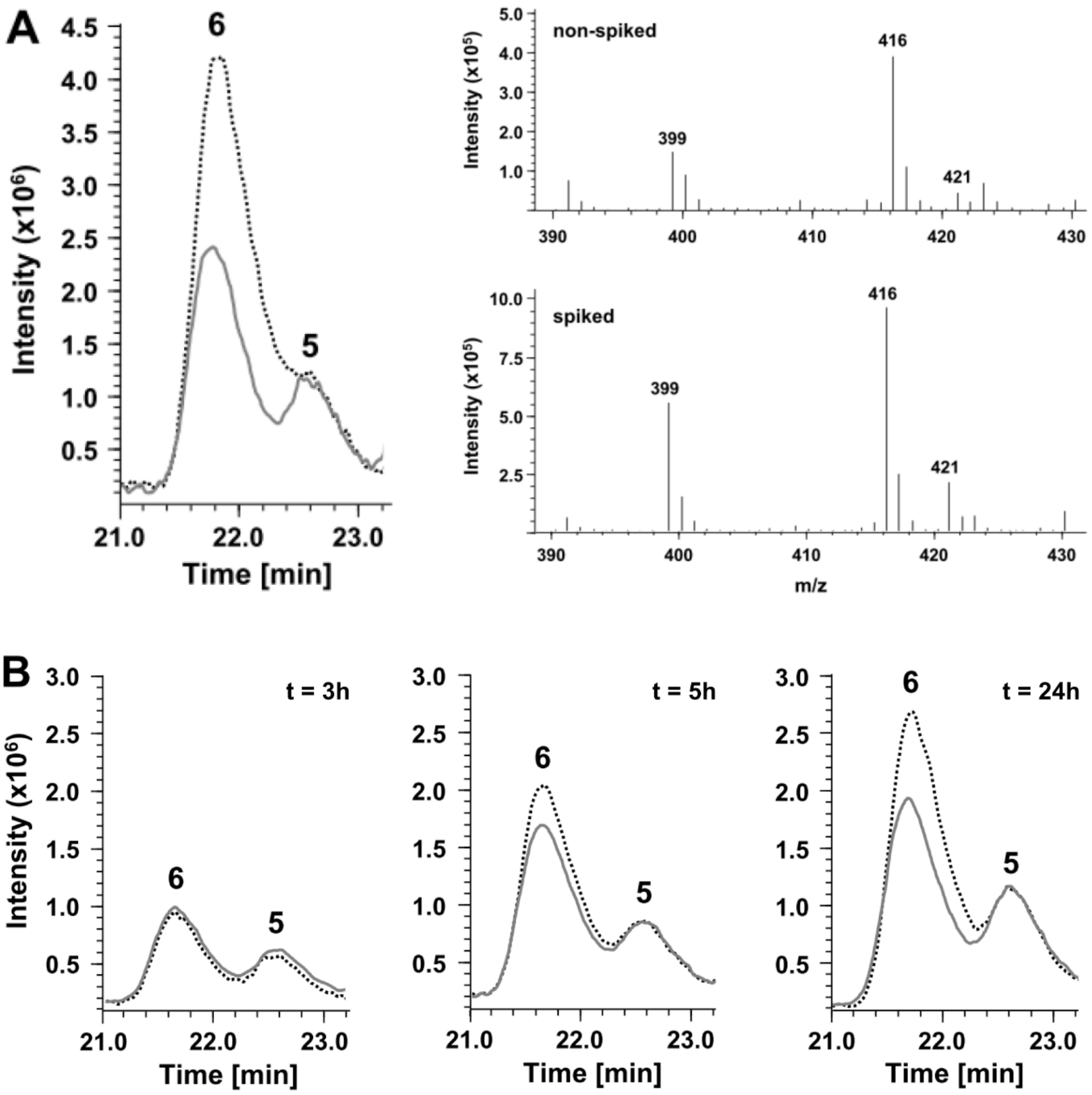

Figure S8. LC/MS analysis (method 4) of one-cell biotransformations of (-)-matairesinol **1** to (-)-deoxypodophyllotoxin **6**. (**A**) LC-chromatogram and m/z-fragmentation at RT = 21.8 min of a reaction sample spiked with 50 µM (-)-deoxypodophyllotoxin **6** (dotted black line) in comparison to the respective non-spiked sample (solid grey line); 399 $[M+H]^+$; 416 $[M+H_2O]$; 421 $[M+Na]^+$. (**B**) (-)-Deoxypodophyllotoxin **6** and (-)-yatein **5** peaks of reaction samples at t = 3 h, 5 h and 24 h with addition of 2.5 mM 2-oxoglutarate at t = 4 h (dotted black lines) in comparison to the respective samples without addition of 2-oxoglutarate (solid grey lines).

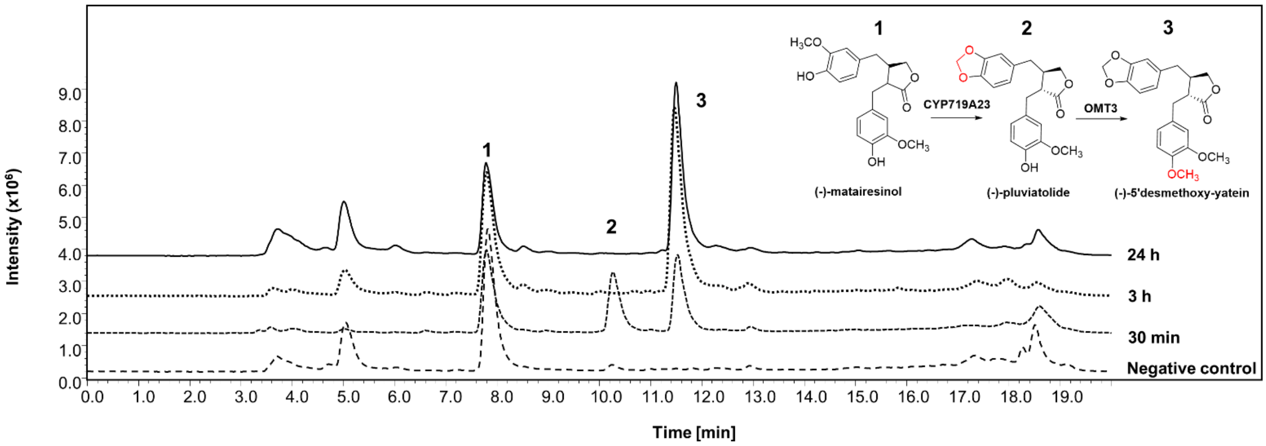

Figure S9. LC/MS analysis (method 1) of OMT3 activity in the cascade reaction with CYP719A23.

A

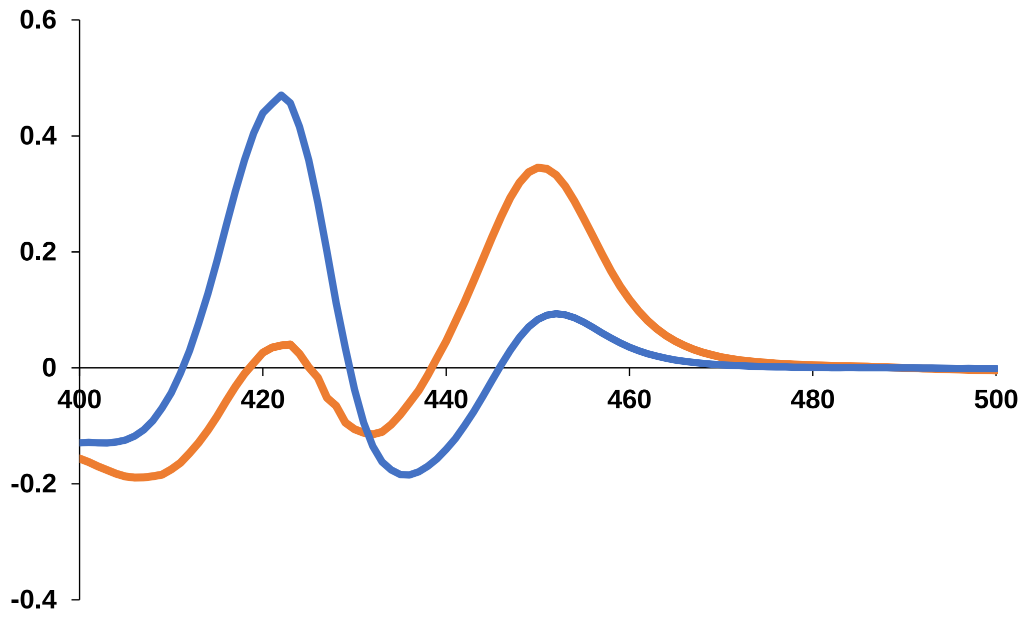

B

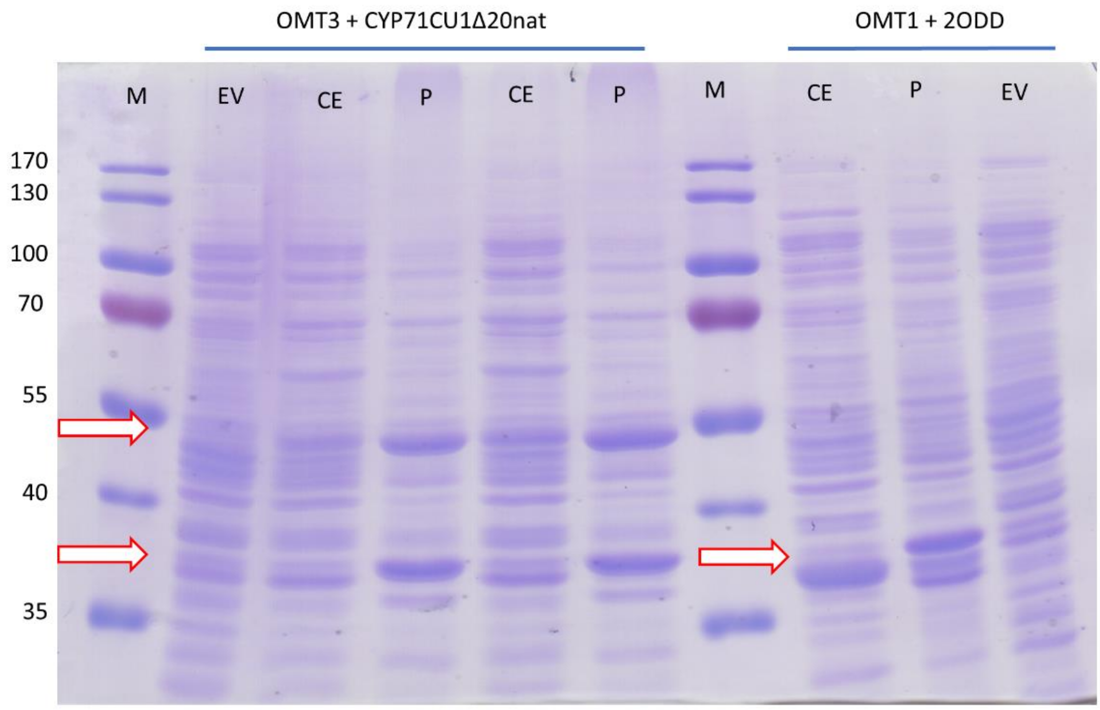

Figure S10. (A) CO-difference spectra of single expressed CYP71CU1Δ20nat (orange line) and CYP71CU1Δ20nat co-expressed with OMT3 (blue line). **(B)** 12.5% SDS-gel of CYP71CU1Δ20nat (54 kDa) co-expression with OMT3 (41 kDa), and OMT1 (38 kDa) with 2-ODD (35 kDa). M: Molecular weight size marker, EV: Vector control without respective genes, CE: Soluble protein fraction after cell disruption, P: Insoluble protein fraction after cell disruption.

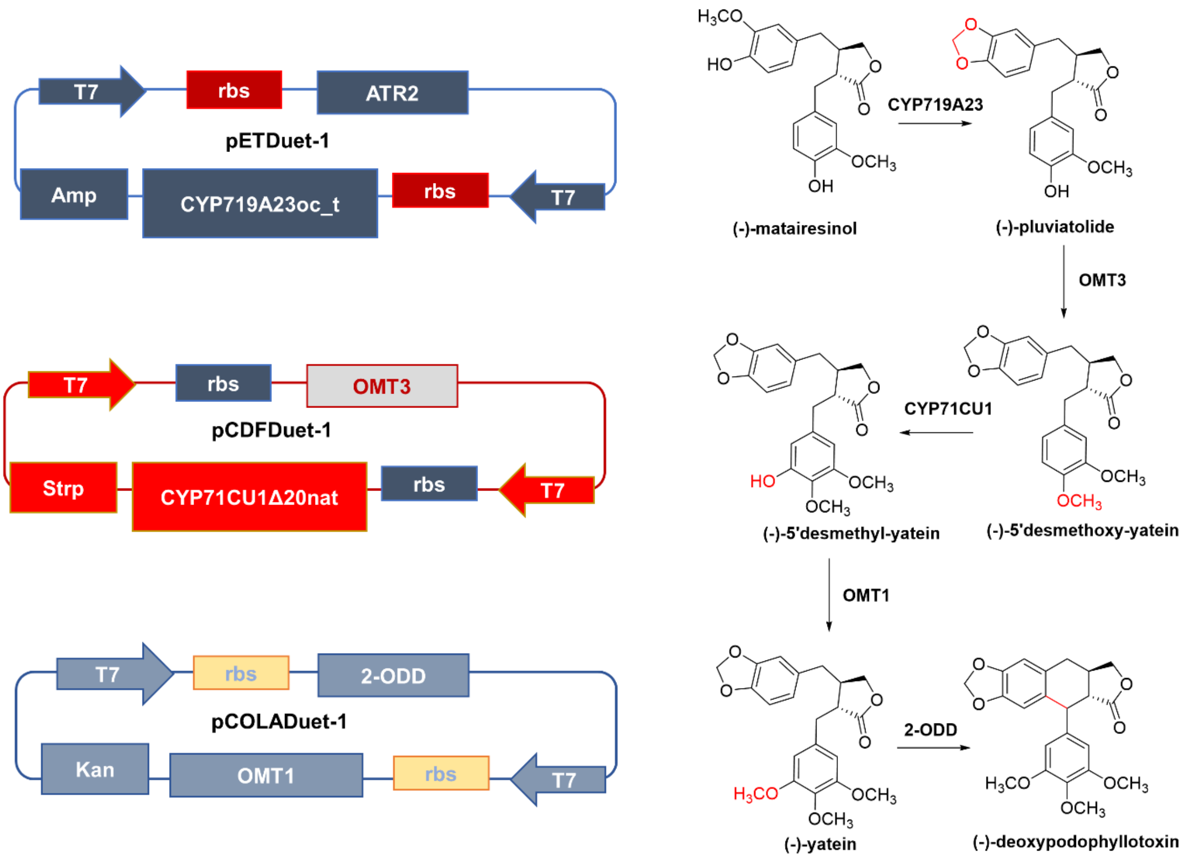

Figure S11. Schematic overview on the plasmid-based modular strategy developed to synthesize (-)-deoxypodophyllotoxin **6** from (-)-matairesinol **1** in *E. coli* via a one-cell 5-steps 6-enzyme reaction cascade.

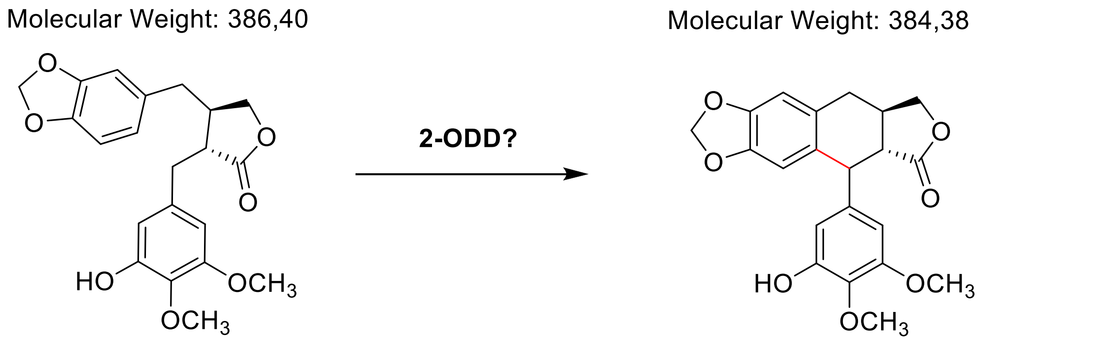

(-)-5'-desmethyl-yatein **(-)-5'-desmethyl-deoxypodophyllotoxin?**

Figure S12. Hypothesized side reaction of 2-ODD occurring in the two-cell approach.

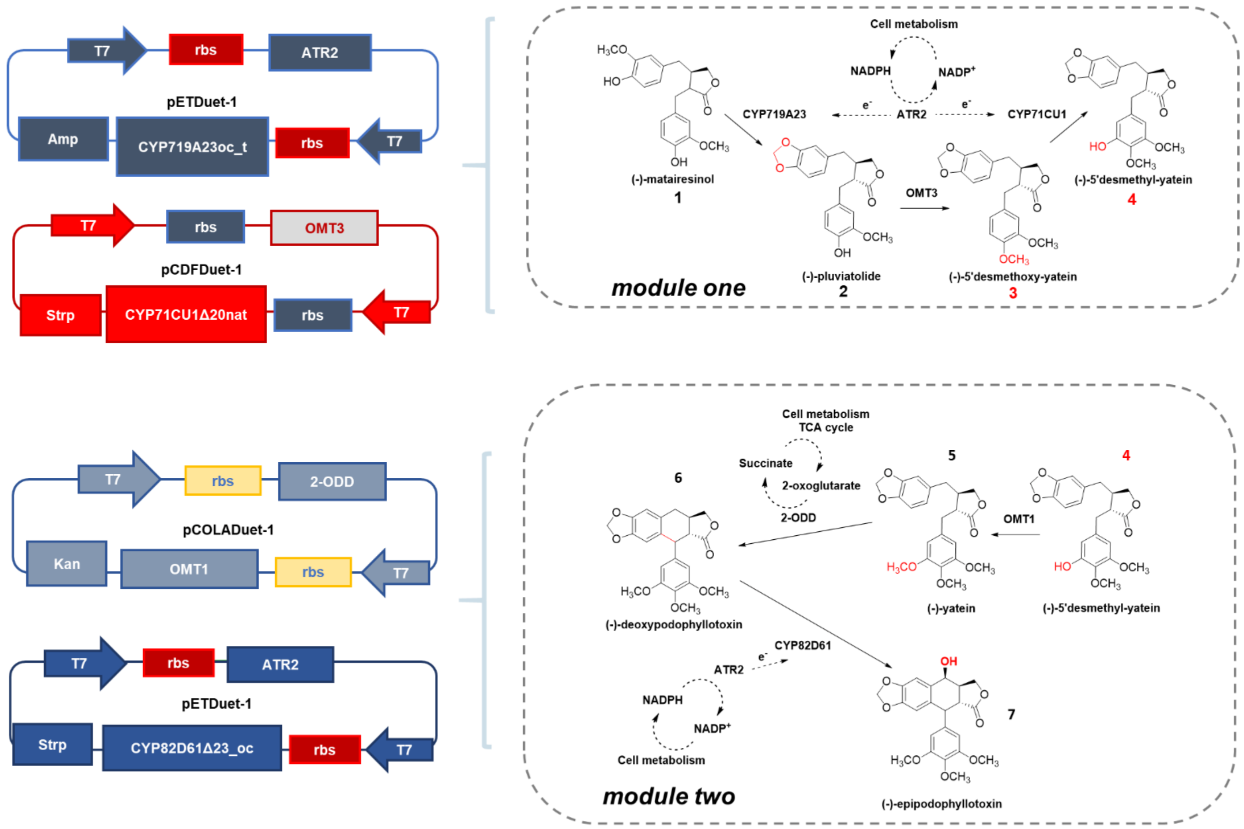

Figure S13 Schematic overview on the plasmid-based modular strategy developed to synthesize (-)-epipodophyllotoxin **7** from (-)-matairesinol **1** in *E. coli* via a two-cell 6-steps 7-enzyme reaction cascade.

4. Supplementary References

1. Omura T, Sato R. The carbon monoxide-binding pigment of liver microsomes I. Evidence for its hemoprotein nature. J Biol Chem. 1964; 239:2370–8.
2. Decembrino D, Ricklefs E, Wohlgemuth S, Girhard M, Schullehner K, Jach G, Urlacher VB. Assembly of plant enzymes in *E. coli* for the production of the valuable (-)-podophyllotoxin precursor (-)-pluviatolide. ACS Synth Biol. 2020; 9:3091-103.
3. Kranz-Finger S, Mahmoud O, Ricklefs E, Ditz N, Bakkes PJ, Urlacher VB. Insights into the functional properties of the marneral oxidase CYP71A16 from *Arabidopsis thaliana*. Biochim Biophys Acta Proteins Proteomics. 2018; 1866:2-10.
4. Lau W, Sattely ES. Six enzymes from mayapple that complete the biosynthetic pathway to the etoposide aglycone. Science. 2015; 349:1224-8.
